# Functional Significance of Calcium Binding to Tissue-Nonspecific Alkaline Phosphatase

**DOI:** 10.1371/journal.pone.0119874

**Published:** 2015-03-16

**Authors:** Marc F. Hoylaerts, Soetkin Van kerckhoven, Tina Kiffer-Moreira, Campbell Sheen, Sonoko Narisawa, José Luis Millán

**Affiliations:** 1 Department of Cardiovascular Sciences, Center for Molecular and Vascular Biology, University of Leuven, Leuven, Belgium; 2 Sanford Children’s Health Research Center, Sanford-Burnham Medical Research Institute, La Jolla, CA, United States of America; University of Dayton, UNITED STATES

## Abstract

The conserved active site of alkaline phosphatases (AP) contains catalytically important Zn^2+^ (M1 and M2) and Mg^2+^-sites (M3) and a fourth peripheral Ca^2+^ site (M4) of unknown significance. We have studied Ca^2+^ binding to M1-4 of tissue-nonspecific AP (TNAP), an enzyme crucial for skeletal mineralization, using recombinant TNAP and a series of M4 mutants. Ca^2+^ could substitute for Mg^2+^ at M3, with maximal activity for Ca^2+^/Zn^2+^-TNAP around 40% that of Mg^2+^/Zn^2+^-TNAP at pH 9.8 and 7.4. At pH 7.4, allosteric TNAP-activation at M3 by Ca^2+^ occurred faster than by Mg^2+^. Several TNAP M4 mutations eradicated TNAP activity, while others mildly influenced the affinity of Ca^2+^ and Mg^2+^ for M3 similarly, excluding a catalytic role for Ca^2+^ in the TNAP M4 site. At pH 9.8, Ca^2+^ competed with soluble Zn^2+^ for binding to M1 and M2 up to 1 mM and at higher concentrations, it even displaced M1- and M2-bound Zn^2+^, forming Ca^2+^/Ca^2+^-TNAP with a catalytic activity only 4–6% that of Mg^2+^/Zn^2+^-TNAP. At pH 7.4, competition with Zn^2+^ and its displacement from M1 and M2 required >10-fold higher Ca^2+^ concentrations, to generate weakly active Ca^2+^/Ca^2+^-TNAP. Thus, in a Ca^2+^-rich environment, such as during skeletal mineralization at pH 7.4, Ca^2+^ adequately activates Zn^2+^-TNAP at M3, but very high Ca^2+^ concentrations compete with available Zn^2+^ for binding to M1 and M2 and ultimately displace Zn^2+^ from the active site, virtually inactivating TNAP. Those *ALPL* mutations that substitute critical TNAP amino acids involved in coordinating Ca^2+^ to M4 cause hypophosphatasia because of their 3D-structural impact, but M4-bound Ca^2+^ is catalytically inactive. In conclusion, during skeletal mineralization, the building Ca^2+^ gradient first activates TNAP, but gradually inactivates it at high Ca^2+^ concentrations, toward completion of mineralization.

## Introduction

Alkaline phosphatases (APs) occur widely in nature, and are found in many organisms from bacteria to man [1, 2]. *In vitro*, APs are quite promiscuous in their substrate specificity, being able to catalyze both the hydrolysis of monoesters of phosphoric acid and a transphosphorylation reaction in the presence of large concentrations of phosphate acceptors [1]; however their *in vivo* functions are quite specific [2]. Four isozymes, with differential tissue expression and encoded by distinct genes, are found in humans: tissue-nonspecific AP (TNAP, also known as liver-bone-kidney type), placental AP (PLAP), germ cell AP and intestinal AP (IAP). Mammalian APs in general, the human isozymes in particular, are homodimeric enzymes and each catalytic site contains three metal ions, two Zn^2+^ (M1 and M2) and one Mg^2+^ (M3), which are perfectly conserved throughout speciation and required for enzymatic activity [3]. An additional metal-binding site M4, that appears to be occupied by Ca^2+^ and is not present in the bacterial enzymes, was revealed upon solving the PLAP 3D structure [4, 5]. This fourth metal site is conserved in all human and mouse APs [6] and presumably represents a novel feature common to many if not all mammalian APs. However, the structural and functional significance of this new M4 metal site remains to be established. Here we have investigated the functional role of this M4 site for TNAP catalysis, an enzyme crucial for skeletal and dental mineralization.

Hypomorphic mutations in *ALPL*, the gene encoding human TNAP (*Alpl* in mice) lead to hypophosphatasia, a heritable form of rickets or osteomalacia. Hypophosphatasia is caused by accumulation of inorganic pyrophosphate (PP_i_), the physiological substrate of TNAP and a potent mineralization inhibitor, in the cartilage and bone extracellular matrix [7, 8]. Thus, a crucial function of TNAP is to hydrolyze PP_i_ in skeletal and dental tissues, restricting the extracellular pool of this mineralization inhibitor [9] and allowing calcification to proceed. In addition, TNAP can also produce phosphate (P_i_) from ATP, which helps drive mineralization in the presence of Ca^2+^ [10]. The current model of the initiation of skeletal and dental mineralization involves crystal formation inside the chondrocyte- and osteoblast-derived matrix vesicles (MVs) favored by P_i_ accumulation resulting from both PHOSPHO1-mediated intravesicular production and transporter-mediated influx of P_i_ produced primarily by the ATPase activity of TNAP. Next, extravesicular calcification is mainly supported by the pyrophosphatase activity of TNAP, and is driven by the availability of P_i_ and Ca^2+^ ions and the presence of a collagenous fibrillar scaffold and guided by other ECM mineral-binding proteins [11]. Early studies indicated that TNAP in cartilage is a Ca^*2+*^ binding glycoprotein [12], but whether Ca^*2+*^ binding occurs at M4 or any other site and whether Ca^*2+*^-binding functionally modulates TNAP activity remains unknown. The overall structure of the M4 site comprises 76 residues (209–285) folded into two β-strands flanked by two α-helices. In PLAP, this region includes a glycosylation site at N249, stabilized by a stacking interaction with W248, and a metal ion coordinated by carboxylates from residues E216, E270 and D285, the carbonyl of F269 and a water molecule, all of which suggest that M4 is occupied by a Ca^*2+*^ ion [4]. Interestingly, hypomorphic mutations of the corresponding residues in TNAP (W253, E218, E274, and D289) cause hypophosphatasia (http://www.sesep.uvsq.fr/03_hypo_mutations.php).

In this report, we have investigated the functional significance of Ca^*2+*^ binding to all four metal ion-binding sites in TNAP to better understand how the activity of TNAP is regulated during skeletal mineralization in an environment with high local Ca^2+^ gradients, further aiming to understand the pathophysiological basis for hypophosphatasia.

## Materials and Methods

### Mutagenesis and expression of TNAP-FLAG and PLAP-FLAG enzymes

Site-directed mutagenesis was performed to generate a series of PLAP and TNAP Ca^2+^-binding site (M4) and peripheral site mutants, using pcDNA3/PLAP-FLAG or pcDNA3.1/TNAP-FLAG vectors as templates, respectively. Site-directed mutagenesis was performed with a Quickchange Site-Directed Mutagenesis kit (Stratagene, San Diego, CA, USA), according to the manufacturer’s instructions, using the oligonucleotide primers listed in Table A in S1 File. COS-1 (ATCC CRL-1650) cells were transfected with plasmids and FLAG-tagged enzymes were collected from the culture supernatant, as described previously [13]. A

### Western Blotting

Culture supernatants of each FLAG-tagged mutant enzyme and the respective native enzymes were purified using an anti-FLAG M2 monoclonal antibody (AbM2) column (Sigma, St Louis, MO, USA) according to the manufacturer’s instructions (the antibody M2 will be referred to as AbM2, to avoid confusion with the metal ion-binding site M2). The protein concentration of each purified sample was determined with a Pierce BCA Protein Assay (Thermo Scientific, Rockford, IL, USA). For Western blots, electrophoresed proteins were transferred to reinforced nitrocellulose membrane (Whatman, Dassel, Germany) followed by blocking in SuperBlock Blocking Buffer in Tris-buffered saline (Thermo Scientific, Rockford, IL, USA). Subsequently, the membranes were incubated with 1μg/ml AbM2, followed by detection as described [14].

### Kinetic measurements

PLAP and TNAP activity were measured as a function of the concentration of the reference substrate p-nitrophenyl phosphate (pNPP; Sigma, St Louis, MO), at the enzyme’s pH optimum in 1 M diethanolamine buffer, pH 9.8, containing 20 μM ZnCl_2_ (Merck, Darmstadt, Germany) and 1 mM MgCl_2_, (Merck) and Lineweaver-Burk plots were constructed to calculate K_m_ and V_max_. From the V_max_ values, k_cat_ was calculated by comparison with V_max_ for a known concentration of native TNAP and historical k_cat_ values [13]. Molar concentrations of p-nitrophenol were calculated, using a molar extinction coefficient ɛ = 18,000 M^−1^cm^−1^, at pH 9.8 (no conversion was made at pH 7.4).

### Functional analysis on the M1-M4 metal sites

Buffers and pNPP substrate were Chelex-treated prior to addition of ZnCl_2_ and/or CaCl_2_ and or MgCl_2_, to minimize contamination with unknown divalent metal ions. Microtiter plates were coated with AbM2 (0.2–0.4 μg/ml) overnight at 4°C, after which plates were blocked with 1% human serum albumin (hSA) for 1 h in Tris-buffered saline (TBS: 50 mM Tris, 137 mM NaCl, 2.6 mM KCl) pH 8.0. TNAP and its mutants were then incubated in TBS, 0.1% hSA, for 3 h at room temperature at various dilutions, taking into account the specific activity for each mutant and the pH at which subsequent analyses would be carried out; dilution factors ranged from 10–200 for the native TNAP solution (stock concentration 20 nM). After washing, plates were subjected to one of the following treatments: 1. Incubation with 1 mM EDTA in TBS, 0.1% hSA, for 2 h; 2. Incubation with CaCl_2_ (0–20 mM) in TBS, 0.1% hSA, up to 16 h; 3. Incubation with 20 μM ZnCl_2_ + 1 mM MgCl_2_ for up to 16 h. After washing, EDTA pre-treated plates were incubated with increasing [ZnCl_2_] (0–40 μM, but mostly 20 μM) to upload bound enzyme, for 2 h. Microtiter plates were then incubated for 60–90 min with the substrate pNPP (1 or 10 mM), dissolved in 1 M Tris-HCL buffer, pH 7.4 [13], containing ZnCl_2_, CaCl_2_ (Merck) and/or MgCl_2_, as specified. Alternatively, pNPP was dissolved in 1 M DEA-buffer, pH 9.8 [13], containing ZnCl_2_, CaCl_2_ and/or MgCl_2_, as specified. The formation of p-nitrophenol was then followed kinetically, via repetitive measurements of A405nm, at 1 or 2 min intervals, up to 90 or 120 min, after which plots of A405nm *vs*. time were constructed. For the indicated reference interval (mostly 60–90 min, where 405nm increased linearly with time), the mean rate of hydrolysis was calculated as *Δm405nm/min*. Acceleration and deceleration of TNAP activity was measured from calculation of *Δm405nm/min* at a given time point, and these slopes were plotted as a function of time, or *vs*. metal ion concentration. Slopes were derived using the GraphPad Prism (San Diego, CA) and represent a measure for the activity of TNAP for the chosen interval. This enzyme kinetics representation was chosen to allow the direct comparison of enzyme activities in conditions where specific activities fluctuated over time. When specified, TNAP bound to microtiter plates was incubated overnight at room temperature for 16h with 250 μM EDTA in TBS, 0.1% hSA, to prepare holo-TNAP.

Two different commercial sources of CaCl_2_ were used in these studies: Calcium chloride dihydrate pro analysi (Merck KGaA, Darmstadt, Germany) with Sr (≤ 0.05%) and Mg (≤ 0.005%) as the most relevant major divalent ion contaminants; and Calcium chloride solution BioUltra, for molecular biology (≈ 1M, Sigma-Aldrich, Saint-Louis, MO), also with Sr (≤ 20 mg/kg) and Mg (≤ 5 mg/kg) as the most relevant major divalent ion contaminants, also containing other divalent ions (≤ 5 mg/kg).

### TNAP protein structure modeling

The primary sequence of human TNAP was submitted to the SWISS-MODEL server [15] to model their tertiary structures, based on homology to human placental alkaline phosphatase (1ZED). The resulting molecular structures for TNAP and its M4 mutants were visualized and analyzed using Chimera v1.7 [16] and Swiss-PdbViewer [17].

### TNAP (mutant) structural analysis

The structural impact of TNAP mutations surrounding M4 was analyzed by antibody mapping and heat inactivation. In the first approach, a TNAP-epitope mapped antibody panel was coated onto microtiter plates, after which a standard concentration of TNAP was added, as previously described [13]. Bound TNAP or TNAP mutant was then detected, using AbM2 [13]. Heat inactivation studies of PLAP and its mutants were performed by incubation at different temperatures, after which remaining PLAP (mutant) activity was measured with pNPP as a substrate [18]. Heat inactivation of the more heat-labile TNAP (mutants) was analyzed by measuring residual activity, after incubation of TNAP (mutant) at 56°C in TBS, as a function of time [13].

### Mathematical model

Binding of Mg^2+^ to the M3 site in TNAP was represented by the following general model:

$$\mathrm{TNAP}+{\mathrm{Mg}}^{2+}\overset{k_{a}}{\underset{k_{d}}{\Leftrightarrow }}\mathrm{TNAP}●{\mathrm{Mg}}^{2+}$$

and K_d_ = K_d_/K_a_ = [TNAP]. [Mg^2+^]/[TNAP●Mg^2+^],

In which k_a_ and k_d_ represent the association and dissociation rate constant of this reaction, respectively and K_d_ is the dissociation constant.

The apparent first order rate constant k_app_ for the binding was calculated from the time to reach 50% of the maximal TNAP activity, t_1/2_ (k_app_ = ln2/t_1/2_) and was then fitted to the equation

$$k_{\mathrm{app}}=k_{a}.[{\mathrm{Mg}}^{2+}]+k_{d}.$$

Plots of k_app_ *vs*. [Mg^2+^] were therefore constructed, to derive k_d_ and k_a_, enabling calculation of K_d_ and comparison to directly determine the K_d_ from dose-response studies. These dose-response curves for TNAP activation *vs*. metal ion concentration were calculated after a steady-state was reached, i.e. from the “reference” interval from 60–90 min, by fitting the data to a one-site binding model (GraphPad Prism), from which plots the K_d_, maximal activity (plateau) and Hill coefficient were derived.

### Statistical analysis

All experiments were carried out at least three times and were confirmed at different enzyme dilutions. When executed in identical conditions, data were averaged and represented as the mean values ± SD; when repeated with different concentrations, one representative example is shown. Dissociation constants are expressed with their SD, calculated form the fitted lines (GraphPad Prism). Groups and dissociation constants were compared using unpaired Student t tests, calculating two-tailed p-values, defined in the text or figure legends, as required.

## Results

### Allosterism for Ca^2+^ binding to Zn^2+^-TNAP

**Fig 1:**
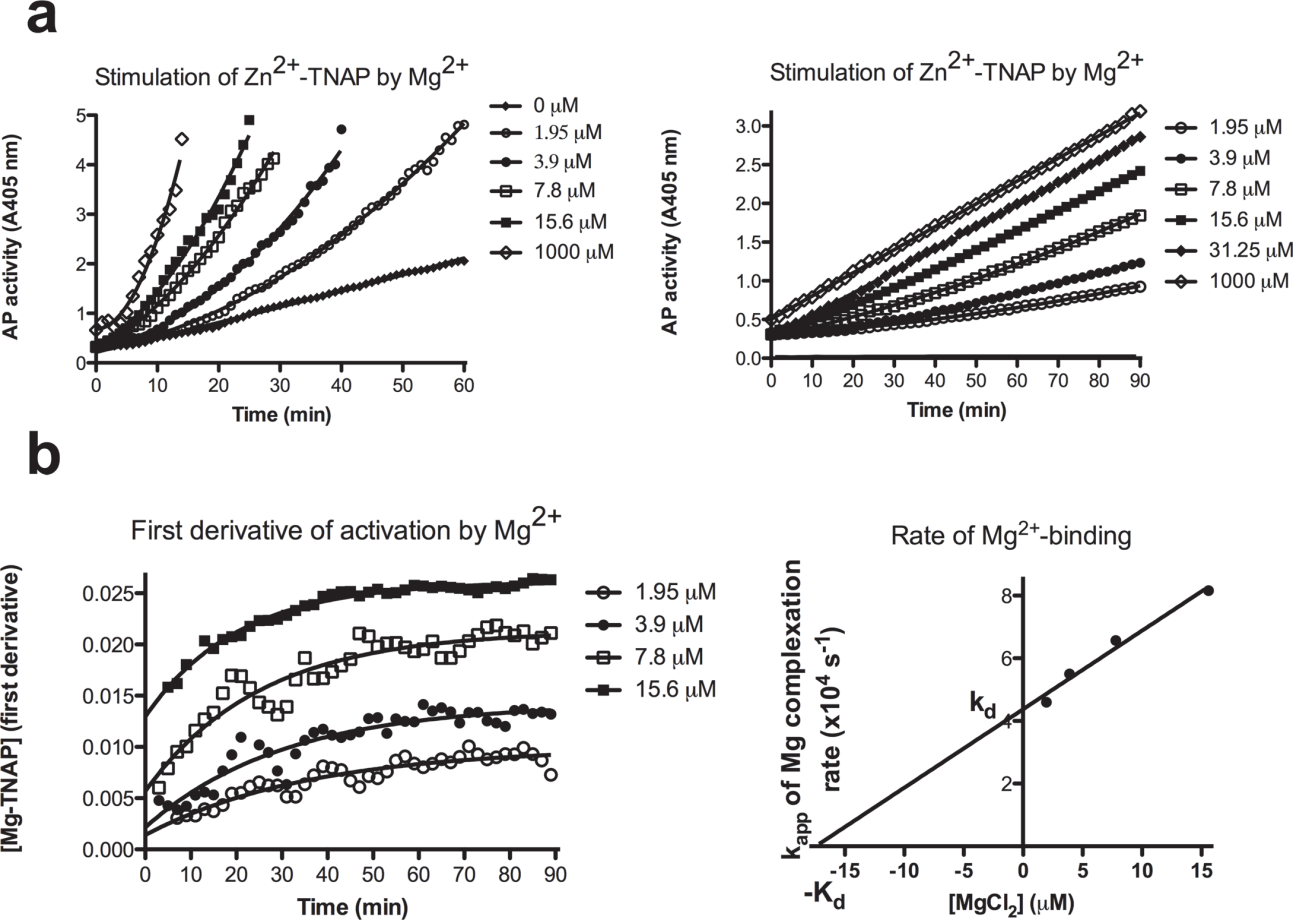
Allosteric activation of Zn^2+^-TNAP by MgCl_2_. a. Progressive AbM2-bound Zn^2+^-TNAP activation, visualized as increasing slopes in plots of A405 nm *vs*. time, for the indicated [Mg^2+^], added to Chelex-pretreated pNPP (10 mM) at pH 9.8 (left panel); repeat of the experiment in (a) at 15-fold lower [TNAP] over a time-interval 0–90 min, for the indicated [Mg^2+^] (right panel); b. Slopes (first derivatives) to the lines in Fig. 1A, right panel *vs*. time, for the indicated [Mg^2+^], describing formation of Mg^2+^/Zn^2+^-TNAP as a function of time (left panel); plots of k_app_ (calculated from Fig. 1B left panel) *vs*. [Mg^2+^] and determination of k_a_ and k_d_ for binding of Mg^2+^ to Zn^2+^-TNAP (right panel);. Experiments representative of at least three replicates with variable enzyme and MgCl_2_ concentrations.

Various lengths of TNAP demetalation and remetalation were tested, prior to selection of a standardized approach. S1 Fig.. illustrates that the demetalation strategy selected in the majority of cases was a compromise resulting in a low residual baseline TNAP activity, but guaranteeing full enzyme recovery upon remetalation with predefined metal ions. Therefore, in these cases, TNAP activity profiles shown were constructed after EDTA-treatment for 2 h, followed by a loading step with 20 μM ZnCl_2_ (yielding Zn^2+^-TNAP with Zn^2+^ in M1 and M2 but free M3). Prior to investigating TNAP activation as a result of binding of Ca^2+^ to M3, we verified whether human TNAP activity complies with the model described for bovine kidney AP [19, 20]. Fig. 1A shows that at high TNAP-concentration (AbM2-bound at 1 nM), increasing concentrations of MgCl_2_ strongly enhanced Zn^2+^-TNAP activity up to 12-fold between 0–1 mM, from baseline (35 mA405nm/min) to a maximum of 417 mA405nm/min, which is similar to the activity observed for native TNAP (non-EDTA treated), measured with 1 mM MgCl_2_ in the pNPP substrate, at pH 9.8 (not shown). Repetition at 15-fold lower TNAP concentration (Fig. 1A, right panel, max. activity 28 mA405nm/min) confirmed strong allosteric TNAP activation by Mg^2+^, but also illustrated the low rate of Mg^2+^-binding to Zn^2+^-TNAP, from the progressive acceleration (i.e. increasing slope) of TNAP activity with time. At high [Mg^2+^] (1 mM), binding was almost immediate (constant slope for ΔA405nm/time). The first derivative of these curves (i.e. the plot of the slopes vs time) describing the formation of Mg^2+^/Zn^2+^-TNAP (plotted and fitted in Fig. 1B, left panel) confirmed this formation to result from a bimolecular reaction, requiring over 90 min to complete for the lowest [Mg^2+^] tested. Calculation of t_1/2_ and corresponding apparent first order rate constants for this reaction, followed by plotting k_app_ *vs*. [Mg^2+^] (Fig. 1B, right panel, r^2^ = 0.977) allowed estimating the kinetic constants of Mg^2+^ binding (k_d_ = 4.4 ± 0.2 x 10^−4^ s^−1^ and k_a_ = 23 M^−1^s^−1^), resulting in K_d_ = k_d_/k_a_ = 17 μM (95% confidence interval: 9.3–39.8), consistent with previously determined values for bovine kidney AP at pH 8 (k_a_ = 7 M^−1^s^−1^ and k_d_ = 4 x 10^–4^ s^-1^). In other words, the allosteric effect of [Mg^2+^] on human TNAP is consistent with that reported for bovine kidney AP [19], with a very slow k_a_ but high affinity (activity measured with 10 mM pNPP, see below). These experiments confirmed that binding of Mg^2+^ stimulated TNAP activity slowly but potently.

**Fig 2:**
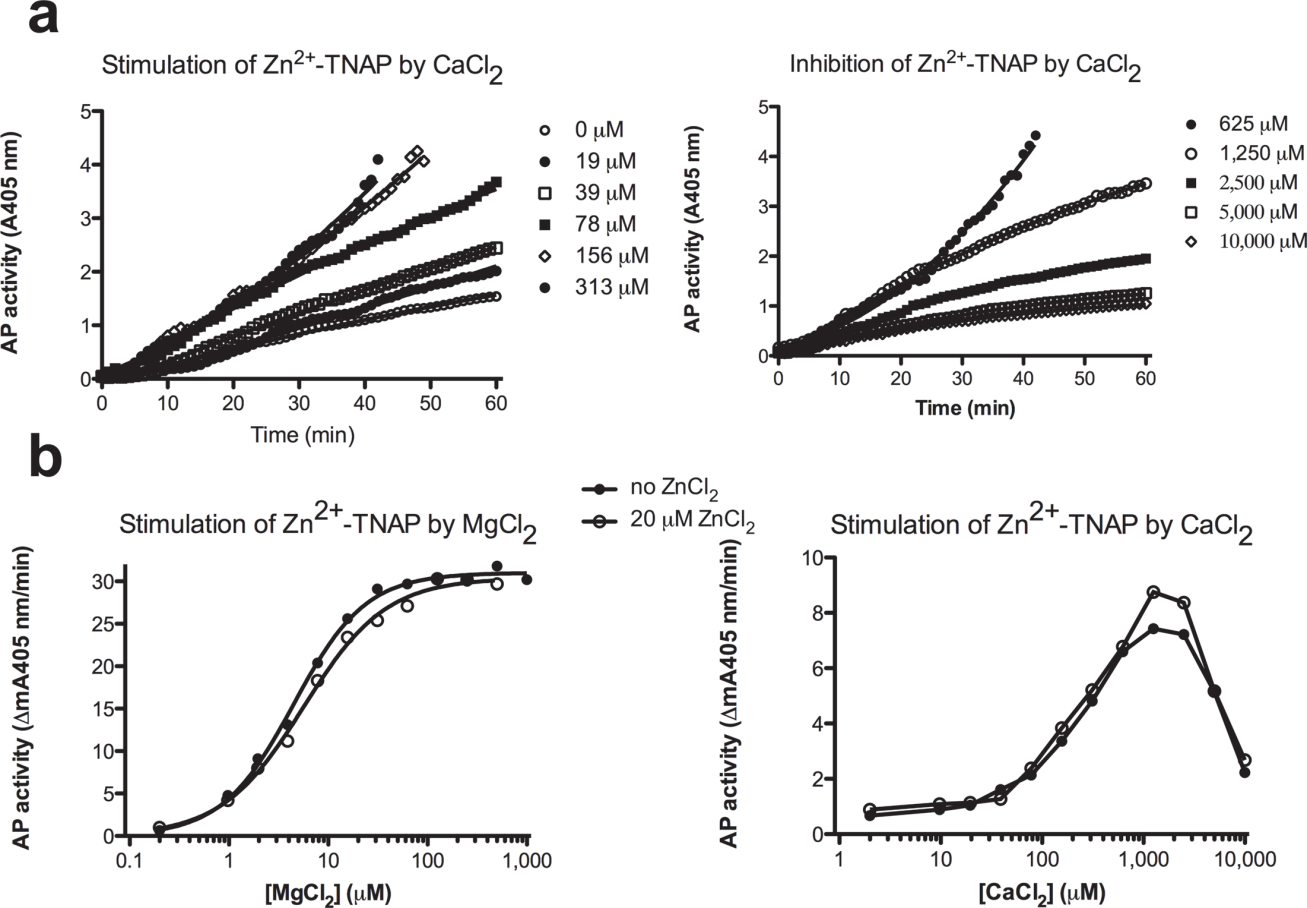
Allosteric activation of Zn^2+^-TNAP by CaCl_2_. a. Progressive AbM2-bound Zn^2+^-TNAP activation, measured as A405 nm *vs*. time, for the indicated [Ca^2+^], added to Chelex-pretreated pNPP (10 mM) at pH 9.8, showing dose-dependent activation (left panel) and inhibition at high concentrations (right panel); b. Dose-response of generated AP activity (mean mA405nm/min) in steady-state (i.e. the slope measured between 60–90 min in Figs. 1A and 2A) for increasing [MgCl_2_] and [CaCl_2_] at identical AbM2-bound [Zn^2+^-TNAP], reflecting the plateau and pseudo-plateau at high [MgCl_2_] and [CaCl_2_] respectively, followed by a steep drop of the TNAP activity in the case of CaCl_2_ (right panel). Activities were measured in Chelex-treated pNPP (10 mM) at pH 9.8, supplemented with MgCl_2_ and CaCl_2_, as indicated. Experiments representative of at least three replicates with variable enzyme and MgCl_2_ concentrations.

Similar incubations as in Fig. 1A (left panel) with increasing [CaCl_2_] also dose-dependently stimulated Zn^2+^-TNAP (Fig. 2A) to a maximal activity of 158 mA405nm/min at 625 μM CaCl_2_, i.e. only 2.6-fold weaker than the maximum for Mg^2+^/Zn^2+^-TNAP in Fig. 1A. However, the binding kinetics (exponential in some cases, although not further analyzed) occurred detectably faster than those for Mg^2+^ binding, at the higher CaCl_2_ concentrations needed to achieve full activation. Fig. 2A, left panel shows functional binding of Ca^2+^ to M3, but high [Ca^2+^] (1.25–10 mM) inhibited Ca^2+^/Zn^2+^-TNAP activity to a level equating 13 mA405nm/min (at 10 mM), i.e. below that of the baseline (22 mA405nm/min). This activity, representing 8% of that of Ca^2+^/Zn^2+^-TNAP (and 3% of that of Mg^2+^/Zn^2+^-TNAP, analyzed at the same [TNAP]), thus abrogated the role of Zn^2+^ in the baseline activity of Zn^2+^-TNAP (Fig. 2A, right panel). Further analysis (S2 Fig..) confirmed that this drop was the result of Ca^2+^ binding to M1 and M2, resulting in the formation of poorly active Ca^2+^/Ca^2+^-TNAP. Analysis at 15-fold less TNAP (as in Fig. 1A, right panel) and plotting the apparent TNAP activity (calculated for the interval range 60–90 min, see S2 and S1 Figs.) *vs*. [MgCl_2_] or [CaCl_2_] (Fig. 2B) revealed a one-site saturation profile for the binding of Mg^2+^ to M3, with an apparent K_d_ = 4.4 ± 0.23 μM and an activity plateau at 31 mA405nm/min, at pH 9.8, using a standard [pNPP] = 10 mM. In the same conditions, the binding of Ca^2+^ followed a biphasic pattern (from baseline activity of Zn^2+^-TNAP), with the ascending limb describing a one-site saturation, with an estimated K_d_ = 220 ± 26 μM and a pseudo-plateau at 8.3 mA405nm/min, i.e. 3.7-fold lower than the Mg^2+^ plateau. The descending limb revealed potent TNAP inactivation with an IC_50_ = 5.4 mM CaCl_2_ and a Hill coefficient = −2.2, compatible with binding to M1 and M2 (see S2 Fig..). The presence of 20 μM ZnCl_2_ in the substrate buffer only displaced the dose-response curves weakly (Fig. 2B), compatible with negligible functional competition between Mg^2+^ or Ca^2+^ and low [Zn^2+^] at M3, at pH 9.8, and essentially confirming that the loading of TNAP with 20 μM ZnCl_2_ allowed the measurement of allosterism in conditions where Zn^2+^-TNAP had been preformed.

**Fig 3:**
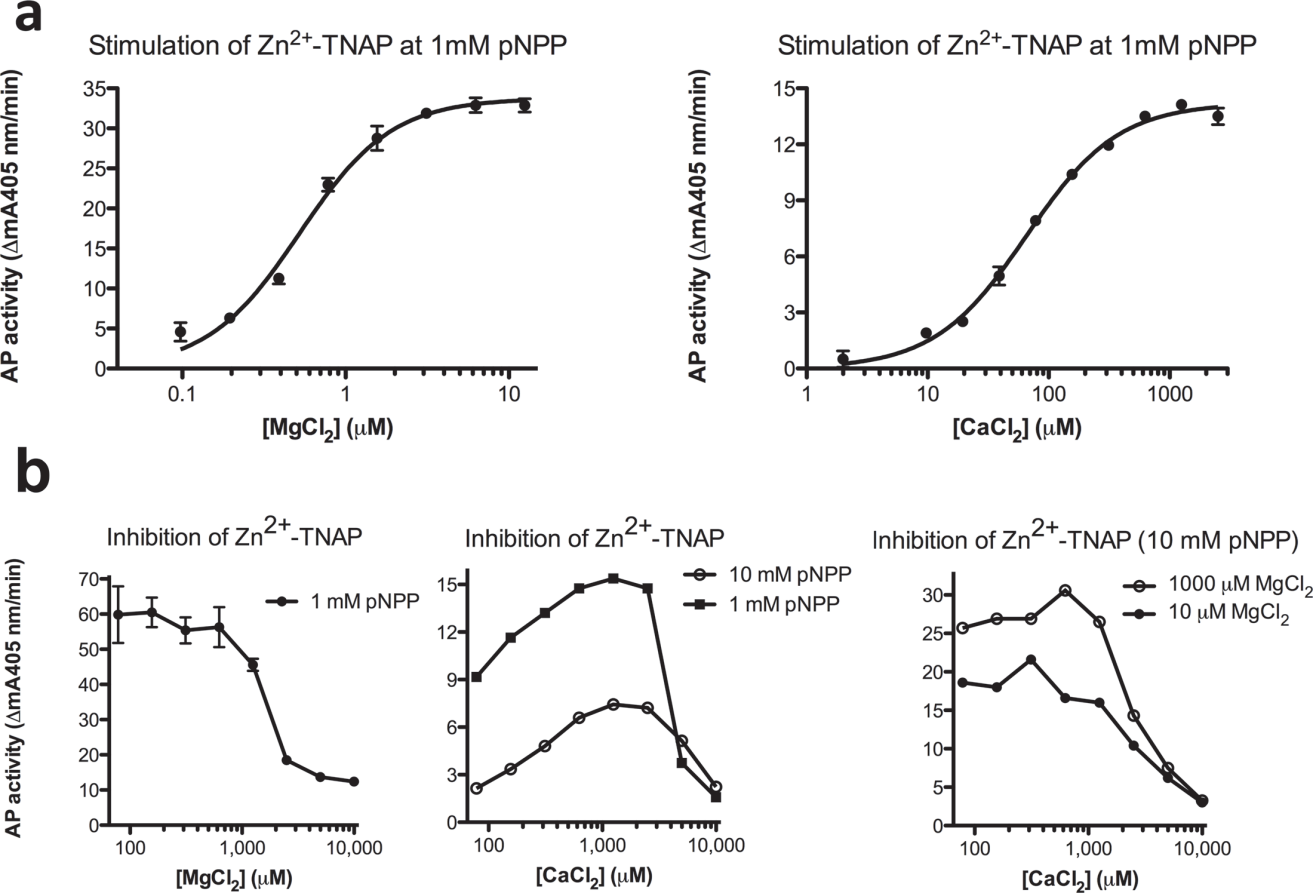
Activation *vs*. inhibition of Zn^2+^-TNAP by MgCl_2_ and CaCl_2_. **a**. Dose-response of generated AP activity (mean mA405nm/min) in steady-state (i.e. the slope measured between 60–90 min) for increasing [MgCl_2_] and [CaCl_2_] at identical AbM2-bound [Zn^2+^-TNAP], measured in Chelex-treated pNPP (1 mM) at pH 9.8; **b**. TNAP inhibition at high [MgCl_2_] and [CaCl_2_], measured in Chelex-treated pNPP (1 or 10 mM as indicated) at pH 9.8, in the presence of the indicated concentrations of MgCl_2_. Results represent mean ± SD for 3 identical experiments or are representative examples of experiments, performed in 3-fold (b, middle and right panel).

To account for complexation between Ca^2+^ (and Mg^2+^) and dissociated phosphate ions in pNPP at pH 9.8 [21], dose-response studies were also repeated at 1 mM pNPP, to reduce the loss of metal ion as a result of its inactivating complexation to pNP-PO_4_^3-^, while taking care to choose sufficiently low enzyme concentrations to not disrupt pseudo-first order conditions (Fig. 3A). M3 saturation by MgCl_2_ occurred with an identical plateau (33.7 mA405nm/min), i.e. did not affect TNAP activity when M3 was saturated with Mg^2+^, but the saturation curve underwent a considerable leftward-shift with K_d_ = 0.52 ± 0.03 μM (8-fold lower as value at 10 mM pNPP, two tailed p<0.0001). Also, M3 saturation by CaCl_2_ (K_d_ = 66 ± 4 μM, with a more accurately determined plateau at 14.2 mA405nm/min) also shifted to lower concentrations (3-fold lower as value at 10 mM pNPP, two tailed p<0.0001), now also reflecting a more precise ratio corresponding to a 2.4-fold lower maximal activity for Ca^2+^/Zn^2+^-TNAP than for Mg^2+^/Zn^2+^-TNAP. High [MgCl_2_] inhibited TNAP activity between 1–10 mM (Fig. 3B, left panel), consistent with the known inhibitory role of Mg^2+^ at M1 and M2 [19] at pH 9.8. Likewise, the descending limbs of the CaCl_2_ curves in Fig. 2B, middle and right panel hardly differed, as a function of the concentration of pNPP or MgCl_2_ in the test tube, excluding the possibility that this drop in activity was determined by the degree of Ca^2+^-pNP-PO_4_^3-^ complexation or the degree of M3 saturation by Mg^2+^ (Fig. 3B, left panel). The activity of Ca^2+^/Ca^2+^-TNAP at 10 mM CaCl_2_ was again about 10% of that of Ca^2+^/Zn^2+^-TNAP and 4.2% of that of Mg^2+^/Zn^2+^-TNAP (Fig. 3B, middle and right panel).

### Functional relevance of the M4 site

**Fig 4:**
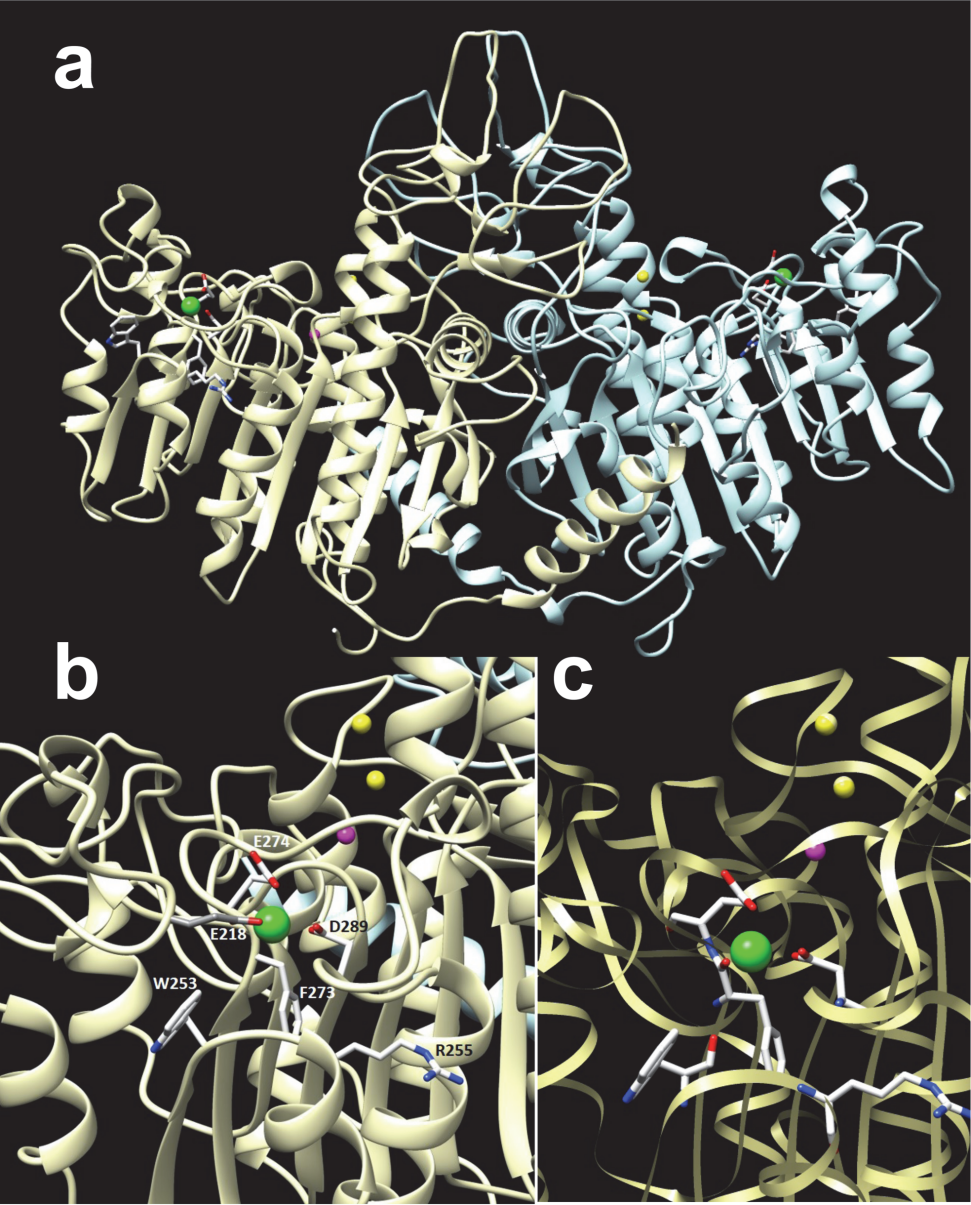
Representation of M4 ligands in the modeled structure of TNAP. Rendering of the 3D structure of the entire TNAP dimer in front view (a) and coordinating residues, detailed in a lateral zoom of the shoulder region harboring M4, in larger detail (b) and in ribbon representation (c).

The crystal structure of PLAP had uncovered a putative Ca^2+^-binding site (M4) in a peripheral location [22], but its significance for AP function remains unknown. To investigate whether binding of Ca^2+^ to M4 also contributes to AP activity, in addition to its binding to M3, a series of site-directed mutants were produced, in which residues potentially coordinating Ca^2+^ in M4 were mutated to alanine in PLAP (where the site was documented) and at the homologous residues in TNAP. Fig. 4A-C displays how those residues (W248, R250, E216, F269, E270, D285 in PLAP; E218, W253, R255, E273, E274, D289 in TNAP) are positioned around the fixed Ca^2+^ ion in a structural homology model of TNAP. Part a in S3 Fig.. shows that all mutants were secreted as FLAG-tagged enzymes. Classical kinetic activity measurements showed that one PLAP mutant and three TNAP mutants were inactive. The remainder of the mutants showed mildly affected kinetic parameters when analyzed via Michaelis-Menten kinetics, using pNPP as substrate without added CaCl_2_ (Table 1). The overall structural impact of most mutations was very limited for the PLAP mutants, with most mutants showing heat inactivation curves comparable to that of reference PLAP (Part b in S3 Fig..). Although TNAP is structurally less stable than PLAP [13], little structural influence on heat stability was noted for the active mutants (Part c in S3 Fig..); in these cases the analysis was done as a function of time at a constant temperature to more gently inactivate the more labile reference TNAP and its mutants.

**Table 1:** Kinetic Parameters of PLAP, TNAP and the M4-site mutants, measured in 1M DEA buffer, pH 9.8, with pNPP as a substrate.

enzyme	K_m_ (mM)	k_cat_ (s^−1^)
	Placental Alkaline Phosphatase (PLAP)	
WT	1.8	460*
E216A	0.9	236
W248A	1.5	165
R250A	1.8	424
F269A	inactive	inactive
E270A	1.1	200
D285A	0.7	18
	Tissue-Nonspecific Alkaline Phosphatase (TNAP)	
WT	0.6	1102*
E218A	inactive	inactive
W253A	0.6	212
R255A	2.3	307
F273A	inactive	inactive
E274A	1.1	384
D289A	inactive	inactive

* based on historical values [13]

**Fig 5:**
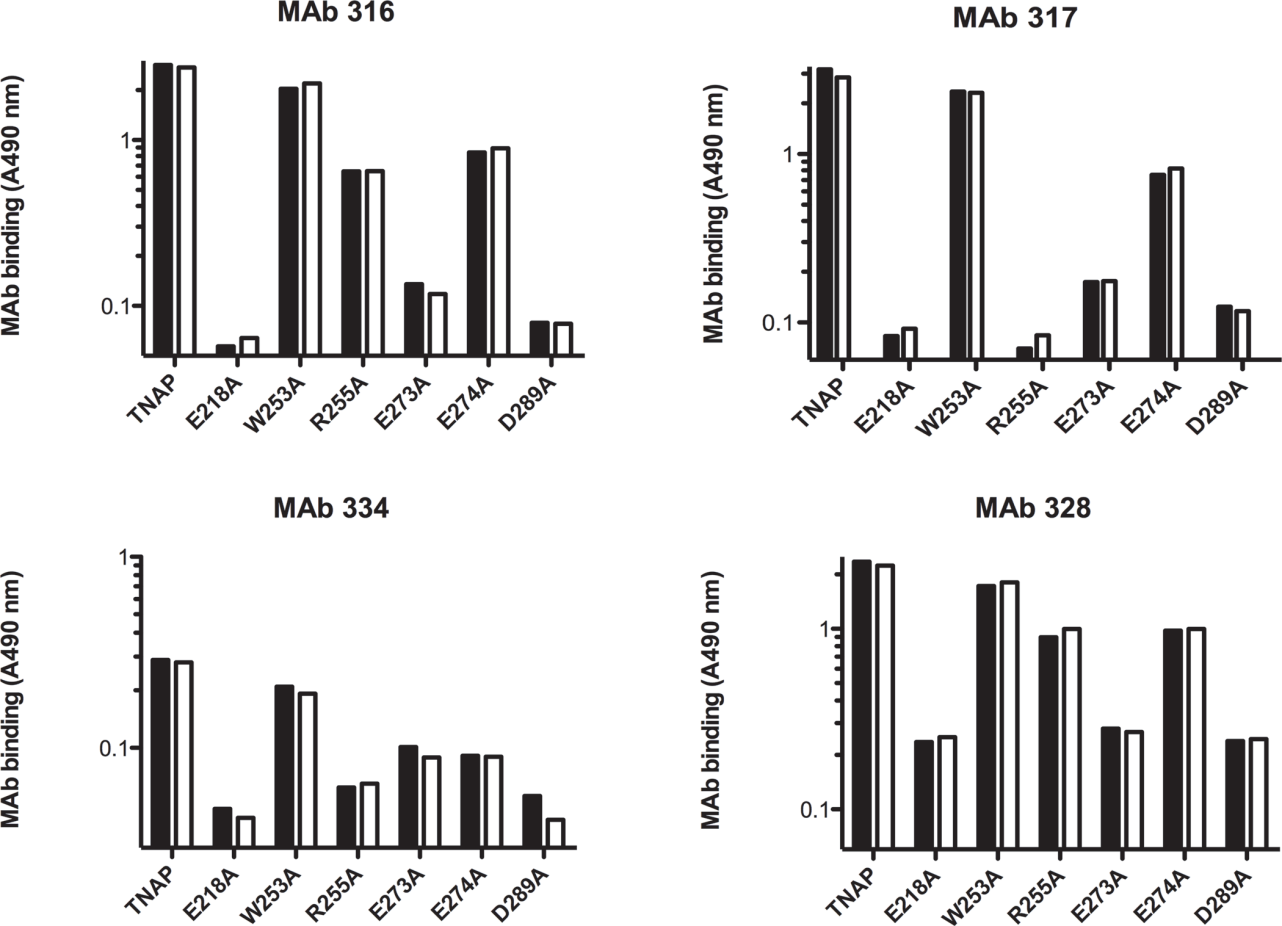
Structural mapping of TNAP M4 mutants. Degree of binding of TNAP and the indicated TNAP mutants to four epitope-mapped monoclonal anti-TNAP antibodies (of 19 studied). Binding was analyzed in the absence (black bars) and presence (white bars) of 1 mM CaCl_2_. TNAP was bound to microtiter plates coated with AbM2 and bound TNAP (mutant) was detected with AbM2, recognizing the FLAG tag.

Structural effects were further investigated both for the active and inactive TNAP mutants, applying an anti-TNAP monoclonal antibody mapping approach using a panel of 19 epitope-mapped antibodies [13], to measure relative affinities in the presence or absence of 1 mM CaCl_2_. Fig. 5 shows that the inactive mutants reacted poorly with the four most discriminating antibodies, indicating that these mutants were not folded properly to maintain a functionally active site. However, this approach could not identify any effect of CaCl_2_ on the affinity of the antibody panel for TNAP or any mutant. Hence, this structural probing, essentially targeting the entire TNAP surface [13] revealed that some mutations perturbed the 3D structure of the resulting TNAP mutants, but that these structural changes occurred independently of the presence of CaCl_2_, as sensed by the anti-TNAP antibody panel.

**Fig 6:**
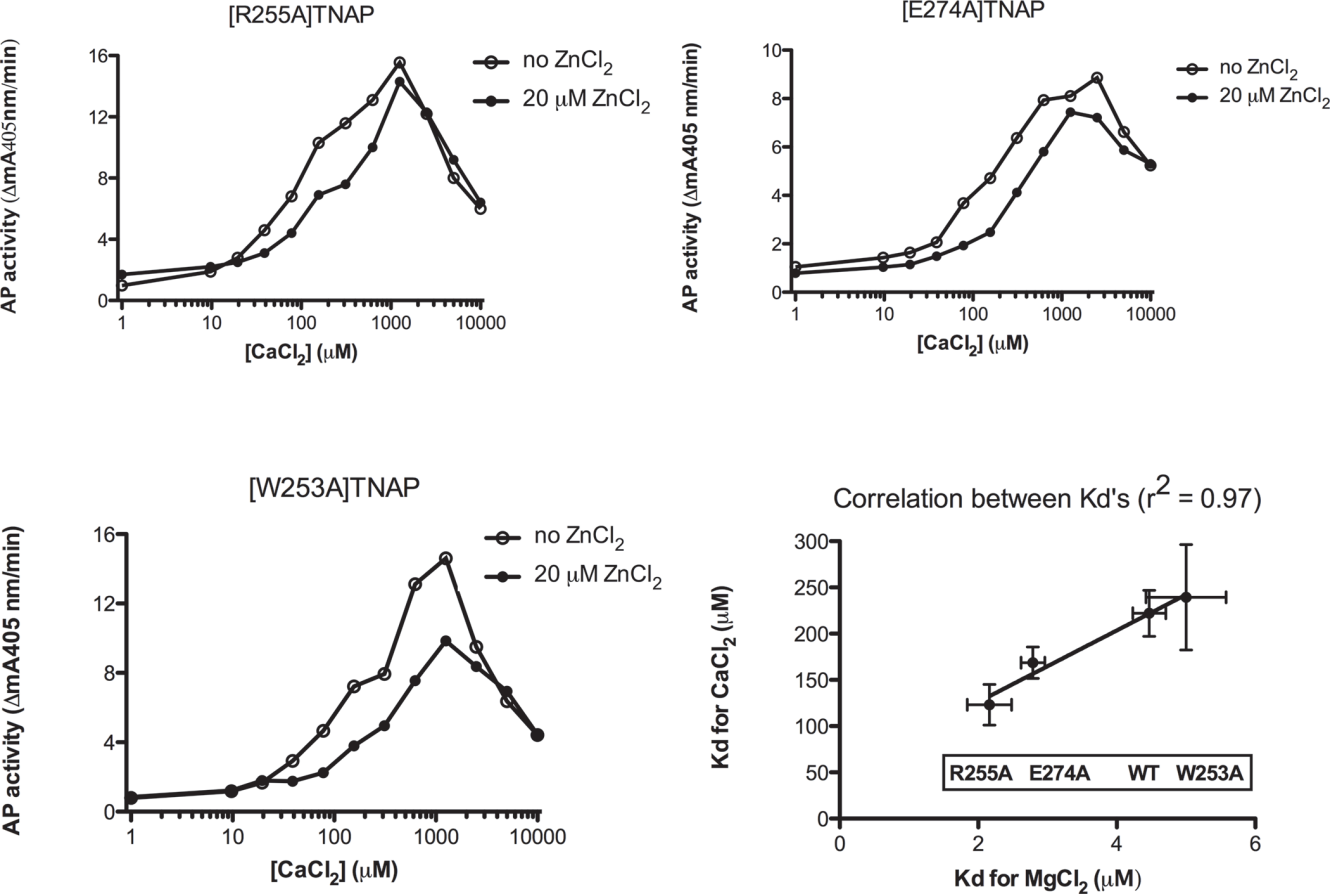
Role of Ca^2+^ binding to M4 in TNAP activity. Dose-response of stimulation of AP activity (mean mA405nm/min) in steady-state (i.e. the slope measured between 60–90 min) at pH 9.8 (10 mM pNPP) for increasing [CaCl_2_], added to AbM2-bound Zn^2+^-TNAP and the indicated mutants, after pre-treatment with EDTA (2 h) and loading with 20 μM Zn^2+^; correlation between calculated apparent K_d_s for the functionally relevant Mg^2+^ and Ca^2+^ binding to TNAP and the three indicated TNAP mutants. Lines constructed in the absence and presence of 20 μM ZnCl_2_ are as indicated; Apparent K_d_s are represented with their respective SD.

To further investigate whether Ca^2+^ binding to M4 contributes to the allosteric activation of TNAP by CaCl_2_, activation of each mutant was analyzed as a function of the concentration of Mg^2+^ and Ca^2+^. The rationale was that M4 mutations would not differentially alter the functional consequences of Mg^2+^ or Ca^2+^ binding to M3, and would impact the overall stimulation by Ca^2+^ only if the M4 site would significantly contribute to TNAP activity, a regulation expected to be defective in at least some mutants. Fig. 6 shows the biphasic Ca^2+^-saturation curves for the three active TNAP mutants. Since the K_m_ for pNPP varies slightly at pH 9.8 for the various mutants, these experiments were conducted at 10 mM pNPP, i.e yielding rather apparent than true K_d_s for the binding of Mg^2+^ and Ca^2+^. Mutations in the active mutants affected the affinity of Mg^2+^ and Ca^2+^ for M3 to some extent, but also that of Zn^2+^ for M3, as evident from the different relative inhibition of TNAP mutants in the presence of 20 μM ZnCl_2_ (Fig. 6). However, the apparent K_d_s measured for Mg^2+^ and Ca^2+^ (in the absence of added ZnCl_2_) correlated strongly (r^2^ = 0.97), showing that Mg^2+^ and Ca^2+^ regulate Zn^2+^-TNAP at the same functionally relevant site, i.e. M3, which was affected by mutations to a comparable degree for both Mg^2+^ and Ca^2+^. These findings identify the TNAP M4 site as a structural determinant, indirectly determining TNAP activity, rather than as a Ca-site directly implicated in the control of enzyme catalysis. As observed above, all M4 mutants were partly inactivated at 10 mM CaCl_2_, an inhibition independent of the presence of 20 μM ZnCl_2_ (Fig. 6).

### Ca^2+^ in TNAP regulation at physiological pH

**Fig 7:**
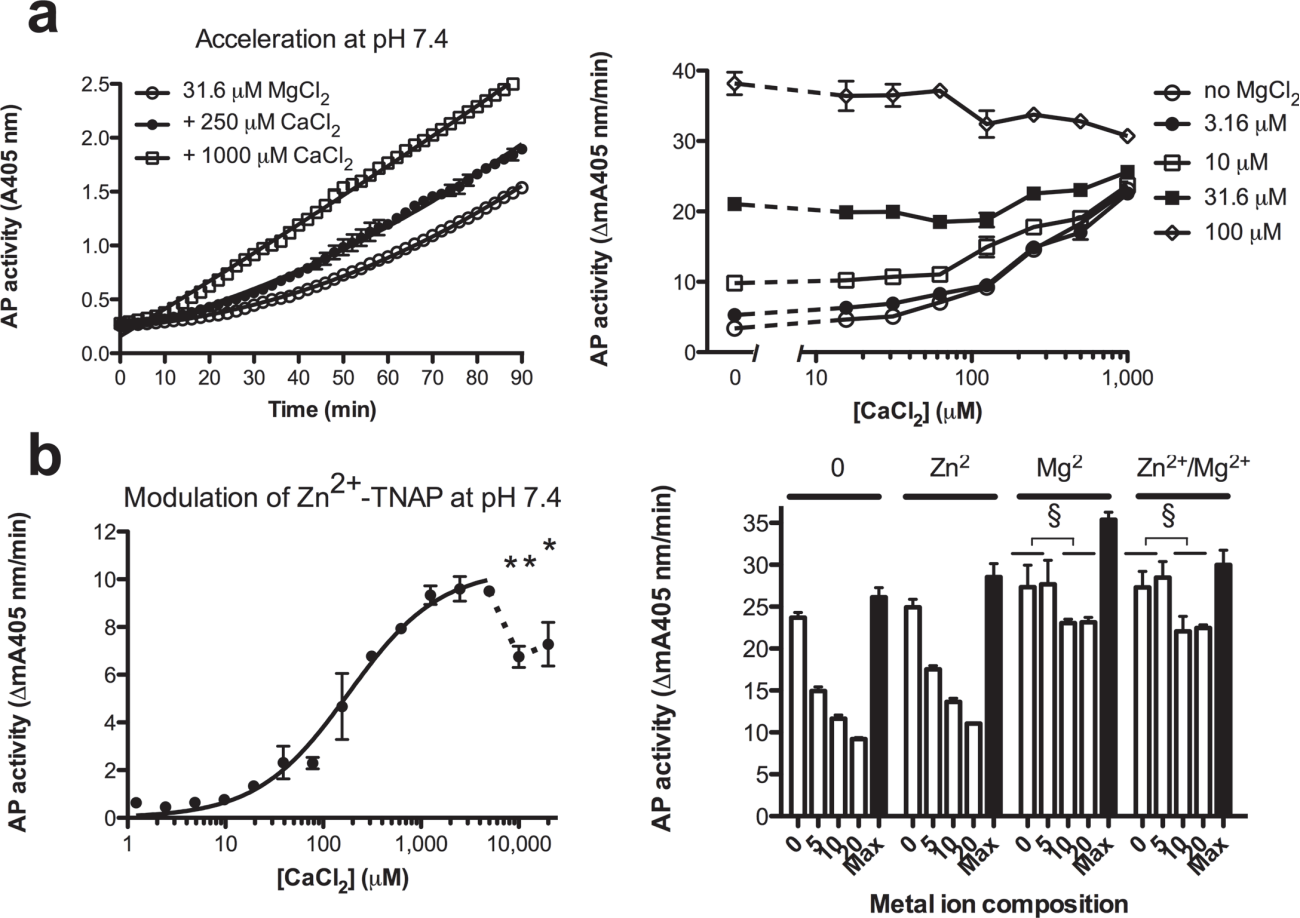
Consequences of Ca^2+^ binding to TNAP at pH 7.4. **a**. Kinetics of TNAP activation by low [Mg^2+^]) and acceleration of activation during formation of Mg^2+^/Zn^2+^-TNAP by increasing [CaCl_2_] as indicated, measured as A405 nm (left panel); additive effects for various combinations of Mg^2+^ and Ca^2+^ during TNAP activation (measured in steady-state, right panel); **b**. Dose-response of activation and partial inhibition of TNAP by CaCl_2_, at the indicated concentrations (left panel); TNAP activity recovery in chelex-treated pNPP, containing the indicated metal ion composition (0: no metal ion; [Zn^2+^] = 20 μM; [Mg^2+^] = 1 mM; Zn^2+^/Mg^2+^ = 20 μM Zn^2+^ + 1 mM Mg^2+^) after 3 h of TNAP binding to AbM2 in the presence of co-incubated CaCl_2_ (0–20 mM, as indicated (right panel); black bars: corresponding activity for maximally active TNAP, (pre-incubation for 3 h in TBS, containing 20 μM ZnCl_2_ + 1 mM CaCl_2_). Results represent mean ± SD for 3 identical experiments. (*p<0.05 and **p<0.01 *vs*. plateau, § p<0.005 vs. [CaCl_2_] = 0).

TNAP activity is routinely analyzed at its alkaline pH optimum, but to properly understand the impact of Ca-homeostasis on the physiological TNAP activity, which is to hydrolyze ATP and pyrophosphate primarily during mineralization [23], we also studied ionized Ca^2+^-interactions with TNAP at pH 7.4. At this pH, coordinating active site residues are more protonated and the pKs describing phosphate dissociation favor preponderance of HPO4^2-^, an ion with a higher solubility product for Ca^2+^ than PO4^3-^, the predominant phosphate ion at pH 9.8 [21]. Since, moreover, the K_m_ for pNPP is very low at this pH, [pNPP] was kept at 1 mM in all cases. Also at pH 7.4, Mg^2+^ binding to M3 is slow and exponential (Fig. 7A, left panel), i.e. Mg^2+^/Zn^2+^-TNAP complex formation is not complete at 90 min, even at [MgCl_2_] = 100 μM). In contrast, binding at M3 is relatively fast for Ca^2+^, reaching steady-state after 10–20 min. In a physiological environment where Ca^2+^ and Mg^2+^ are both present, they display additive effects on M3 during activation of Zn^2+^-TNAP. As a consequence of its faster binding at pH 7.4, Ca^2+^ has a relative competitive advantage during binding, 1 mM CaCl_2_ capable of enhancing the activity of forming Mg^2+^/Zn^2+^-TNAP, due to faster Ca^2+^-binding to M3 (Fig. 7A, left panel), Fig. 7A, right panel further illustrates the additive interplay of both metal ions on Zn^2+^-TNAP activation at pH 7.4, illustrating their near-equivalence.

At pH7.4, TNAP was dose-dependently activated by CaCl_2_ (Fig. 7B, left panel). From the ascending limb a K_d_ = 217 ± 50 μM was calculated for the binding of Ca^2+^, with a plateau activity of 11.1 mA405nm/min. This value differed from K_d_ = 66 ± 4 μM, determined at pH 9.8 by a factor 3 only (two tailed p<0.0001). Similar plots of TNAP saturation by Mg^2+^ (Part a in S4 Fig.., left panel) yielded a K_d_ = 82 ± 27 μM for the binding of Mg^2+^, i.e. considerably higher than K_d_ = 0.52 ± 0.03 μM determined at pH 9.8 (two-tailed p<0.0001) with a plateau activity of 27.4 mA405nm/min. In conclusion, at M3 Ca^2+^ is almost equipotent to Mg^2+^ at pH 7.4, with plateau activities again differing 2.5-fold. Yet, some TNAP inactivation was noted at 10 and 20 mM CaCl_2_ (Fig. 7B, left panel), milder than at pH 9.8, but not absent.

### Ca^2+^-mediated loss of activity at pH 7.4

Fig. 7B, right panel shows that the binding of TNAP to AbM2 in the presence of 5–20 mM CaCl_2_ for 3 h, resulted in a dose-dependent 2.5-fold reduction of TNAP activity at 20 mM, when subsequently measured in Chelex-treated pNPP, without further metal ions. ZnCl_2_, added to the pNPP substrate hardly affected the activity measured, but added MgCl_2_ recovered activity fully after pre-incubation with 5 mM CaCl_2_ and for about 80% after pre-incubation with 10 and 20 mM CaCl_2_ (p<0.005 for 10 and 20 mM combined *vs*. 0 mM). These experiments illustrated that CaCl_2_ readily displaced M3-bound Mg^2+^, reducing TNAP activity 2.5-fold, as expected, a loss easily recovered by Mg^2+^ added to the pNPP substrate.

**Fig 8:**
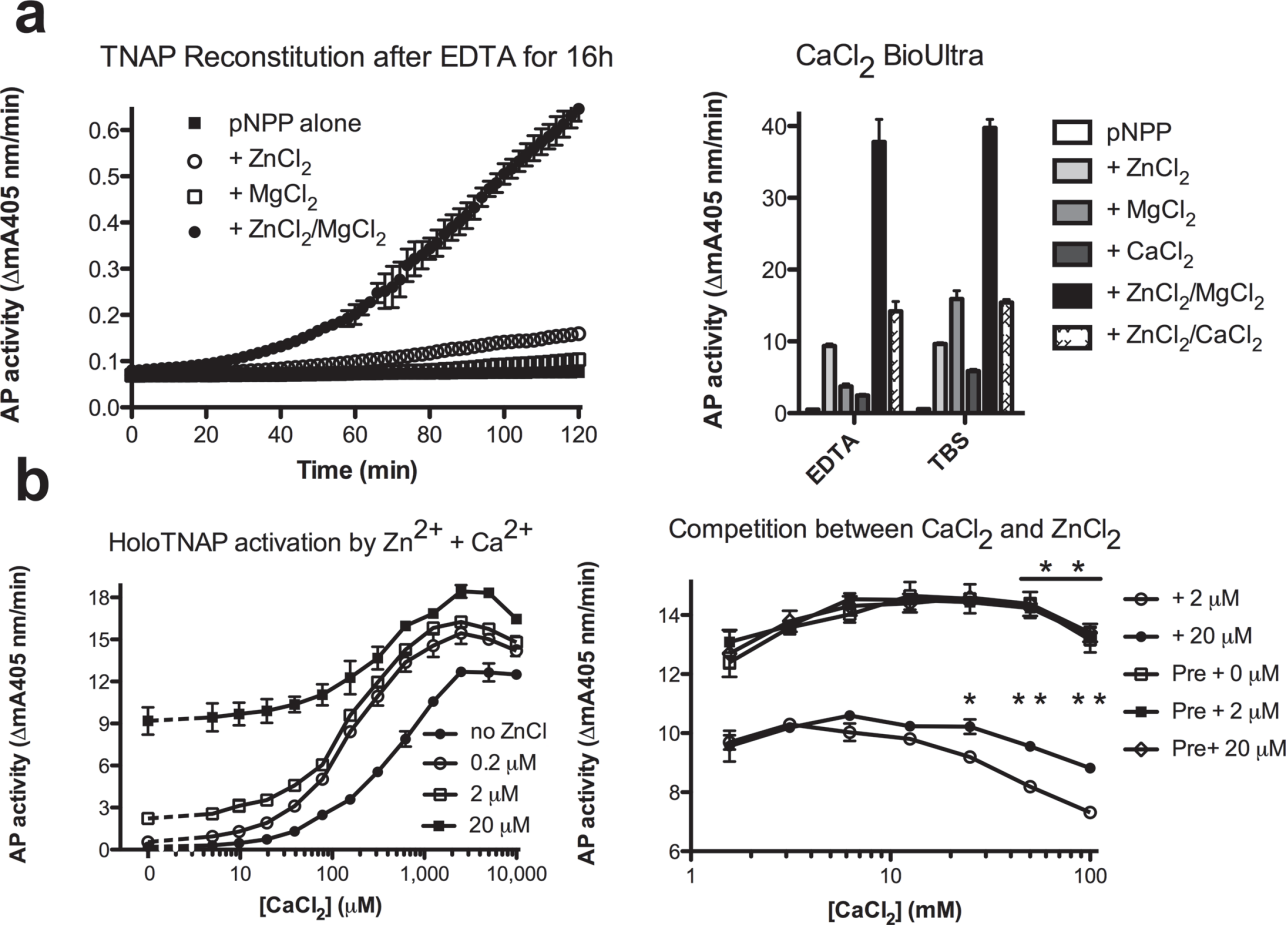
TNAP inactivation by high [CaCl_2_] at pH 7.4. **a**. Reconstitution of TNAP activity by 20 μM Zn^2+^ + 1 mM Mg^2+^, but not by the individual metal ions, added to fully demetalated holo-TNAP, starting after 60 min (left panel); comparison of specific TNAP activity of (holo)-TNAP, after reconstitution with the indicated metal ion composition, after overnight treatment with TBS, with or without added EDTA (250 μM); b. Dose-dependency of holo-TNAP reconstitution by CaCl_2_ (0–10 mM), in the absence or presence of the indicated [ZnCl_2_] (0–20 μM, left panel); competition between the indicated concentrations of Zn^2+^ (2 μM and 20 μM) and increasing concentrations of CaCl_2_; minor displacement of TNAP-bound Zn^2+^ (overnight Zn^2+^ preloading indicated as “Pre”) by increasing concentrations of CaCl_2_, independently of the presence of free Zn^2+^.(0–20 μM). Results represent mean ± SD for 3 identical experiments (*p<0.003, **p<0.0001,).

However, the irreversible loss of TNAP (20%) at higher [Ca^2+^] (10–20 mM) was compatible with some Ca^2+^-induced TNAP inactivation. To enable proper study of the interaction of Ca^2+^ and M1 and M2, we have prepared holo-TNAP, by overnight incubating AbM2-bound TNAP with 250 μM EDTA, at room temperature. This procedure fully stripped TNAP from its bound metal ions (Fig. 8A, left panel), resulting in complete TNAP inactivation, as measured with chelex-treated pNPP, and showing minor enzyme activity upon inclusion of 20 μM ZnCl_2_ in the substrate. Whereas 1 mM MgCl_2_ did not cause activation, combined, ZnCl_2_ + MgCl_2_ reconstituted TNAP over 1 h to over 80% of its initial activity. Fig. 8A, right panel shows the calculated enzyme activities for reconstituted TNAP, measured in different conditions. Overnight incubations with chelex-treated TBS, lacking EDTA were less efficient, confirming the presence of low residual TNAP-bound Zn^2+^, acting in concert with MgCl_2_ in the substrate.

Fig. 8B, left panel shows that [CaCl_2_] unexpectedly triggered holo-TNAP activation dose-dependently, with an apparent K_d_ = 509 ± 49 μM. To quench this non-specific TNAP activation, triggered by divalent metal ion contaminants [24], we performed further dose-response studies in the presence of known concentrations of Zn^2+^ (0.2–20 μM). This procedure shifted Ca-saturation curves to lower [CaCl_2_] with K_d_ = 144 ± 49 μM for 0.2 μM and K_d_ = 137 ± 24 μM for 2 μM, i.e. in fair agreement with K_d_ = 217 ± 50 μM, for the binding of CaCl_2_ to Zn^2+^-TNAP at M3 (Fig. 7B, left panel).

Therefore, to analyze the competition between Ca^2+^ and Zn^2+^ *vs*. Ca^2+^-induced Zn-displacement from M1 and M2, first AbM2-bound holo-TNAP was incubated with high [CaCl_2_] in the presence of 2 or 20 μM ZnCl_2_ (thus quenching non-specific TNAP activation by divalent metal ions in the CaCl_2_ solutions). Fig. 8B, right panel confirms that CaCl_2_ competes with ZnCl_2_ for binding to M1 and M2 at [CaCl_2_]>10 mM, more efficiently when [ZnCl_2_] is low (2 μM), with an apparent IC_50_ around 250 mM. On the contrary, when AbM2-bound TNAP was fully loaded overnight with 20 μM ZnCl_2_, CaCl_2_ only displaced bound Zn^2+^ at concentrations as high as 100 mM, independently of the presence of soluble ZnCl_2_ (0–20 μM).

## Discussion

APs are zinc metalloenzymes, with Zn^2+^ binding to M1 and M2, and allosterically activated by Mg^2+^ binding to M3 [4, 6]. Whereas substitution of Zn^2+^ by most metal ions, except Co^2+^ inactivates enzyme activity, allosteric activation of APs can also be provided at M3 by other ions like Mn^2+^, Co^2+^, Ni^2+^, including Ca^2+^ [25]. Because matrix vesicle-induced mineralization is a process that requires the generation of reaction products by TNAP, the present work was undertaken to study TNAP functionality during exposure to an increasing Ca^2+^ gradient, including the mechanism of TNAP inhibition, at high [CaCl_2_] [26, 27]. Inspired by the existence of a fourth metal ion site, occupied by Ca^2+^ [4], we have presently investigated contributions by all four metal sites. We found that Ca^2+^, by binding to M3 is a fairly good allosteric activator of TNAP when bound to M3, but that binding at M4 hardly influences the catalytic activity of TNAP. A strong determinant of TNAP activity is the availability of Zn^2+^, free Zn^2+^ being competed out by high Ca^2+^ concentrations and TNAP-bound Zn^2+^ being displaced from M1 and M2 at still higher Ca^2+^ concentrations, both resulting in virtual TNAP inactivation. Elegant zinc mapping studies in osteons [28] have revealed co-distribution of alkaline phosphatase with zinc at the calcification front, providing an explanation for the long-lasting presence of Zn^2+^-TNAP in bone matrix.

In humans, baseline plasma Zn^2+^ concentrations average around 12 μM [29] and free plasma Mg^2+^ averages 0.4–0.6 mM, with free Ca^2+^ averaging 1.1–1.3 mM [30]. The present affinity determinations at physiological pH therefore predict circulating TNAP is properly charged at M1 and M2 with Zn^2+^ and is saturated at M3 primarily with Mg^2+^. However, in an environment where matrix vesicles generate a gradient of Ca^2+^ during early mineralization and TNAP generates P_i_ from ATP, PP_i_ and other physiological substrates, the relative balance between divalent metal ions as found in plasma will gradually be disturbed by gradients of P_i_ and PP_i_, inducing formation of poorly soluble hydroxyapatite. Our present findings confirm at pH 7.4 that Ca^2+^ and Mg^2+^ are quite complementary in the allosteric activation of TNAP. Thus, a relative drop of [Mg^2+^] is not a matter of concern, since transported Ca^2+^ is capable of adequately substituting for Mg^2+^. Indeed, at physiological pH, the affinities of Ca^2+^ and Mg^2+^ for M3 only differ 2–3 fold and the maximal activity is only 2.5 fold weaker for Ca^2+^/Zn^2+^-TNAP than for Mg^2+^/Zn^2+^-TNAP.

Mg^2+^ can also easily be replaced at M3 by Mn^2+^, Co^2+^ and Ni^2+^ [19, 25], but our present findings confirm that Mg^2+^ does not generate activity when incubated with the apoenzyme, as a result of binding to M1 and M2 [19]. At physiological pH, TNAP has a low K_m_ for common substrates [13, 18] or relative catalytic efficiency comparisons between different activity states are dictated by the catalytic rate constants primarily. TNAP has a 50-fold lower k_cat_ for pNPP at pH 7.4 than at pH 9.8. Correspondingly, also the affinities of catalytically active metal ions differ at both pHs and physiologically relevant comparisons for metal ion substitutions in the TNAP active site can only be made representatively at pH 7.4. Correspondingly, we presently found that Ca^2+^ binds to M1 and M2, rapidly at pH 9.8, but more slowly at pH 7.4, a process completing dissociation of bound Zn^2+^, a slow process, because of the high affinity of Zn^2+^ [20]. Hence, the main conclusion of our present work is that TNAP is extremely robust in a Ca^2+^-rich (patho)-physiological environment. The rapid substitution of Mg^2+^ for Ca^2+^ in M3 hardly results in any loss-of function. The slow substitution at pH 7.4 of Zn^2+^ for Ca^2+^ as a result of competition or Zn-displacement at M1 and M2 generates an enzyme (Ca^2+^/Ca^2+^-TNAP) 20-fold less active as the parent Mg^2+^/Zn^2+^-TNAP and still 10-fold less active as Ca^2+^/Zn^2+^-TNAP.

We have noted before that specific amino acid substitutions affecting catalysis at pH 9.8 did not have a similar effect at physiological pH [18, 31]. Yet, the relative residual activity, measured for Ca^2+^/Ca^2+^-TNAP at pH 9.8 and pH 7.4 are comparable. On a relative scale, on which Mg^2+^/Zn^2+^-TNAP is 100% active at pH 9.8, Ca^2+^/Zn^2+^-TNAP is 40% active and Ca^2+^/Ca^2+^-TNAP is 5% active. On that same scale, at pH 7.4, Mg^2+^/Zn^2+^-TNAP is 2% active and Ca^2+^/Zn^2+^-TNAP is 0.8% active, Ca^2+^/Ca^2+^-TNAP extrapolated to be virtually inactive. From a physiological perspective, calcium incorporation in TNAP does not destroy TNAP, but the relative balance between ionic calcium, P_i_, PP_i_, other divalent ions and the relative availability of Zn^2+^ during synthesis of TNAP are all crucial factors, determining proper charging at M1 and M2 during long exposure to high Ca^2+^ concentrations.

We found that the impact of Ca^2+^ on TNAP activity could be explained entirely by its interactions with M1–3. In contrast, contributions by M4 were structural. Mutations of several TNAP ligands coordinating the M4 binding site did affect the conformation of the resulting mutants to a variable degree, from minor effects for some mutants to complete loss of the 3D-structure for others, as concluded from combined epitope analysis by an antibody panel of 10 antibodies, heat inactivation studies and classical kinetic analysis. Compared to native TNAP, some mutants manifested a mildly influenced affinity for Mg^2+^ binding to M3, indicative both of gain-of-function, as well as loss-of-function. The relative effects on the affinity for Ca^2+^ were very similar, i.e. the respective K_d_s for Mg^2+^ and Ca^2+^ correlated well for the various mutants and native TNAP. These TNAP M4 mutants manifested slight changes in their allosteric properties, which could fully be explained by the allosteric properties of M3, i.e. we did not find any evidence for a role of M4 in catalysis. Instead, our structural analyses identified some ligands of the M4 site to be critical structural elements of TNAP which, despite their distant location from the active site have a dominant role on the active site integrity when mutated to residues such as encountered in some hypophosphatasia patients (http://www.esep.uvsq.fr/03_hypo_mutations.php). Yet, we did not observe any structural change for the anti-TNAP monoclonal antibody panel, when affinities were measured in the absence or presence of 1 mM CaCl_2_, despite detection of primary changes in 3D-structure in the TNAP mutants. These findings also rule out that Ca^2+^ binding to M4 will participate in structural folding of the M4 ligand area.

We have previously described APs as allosteric enzymes in which asymmetry between monomers generates activity patterns which differ from the expected weighed properties of the two monomers [32]. In particular, negative cooperativity can be generated between both monomers, when they are differently metalated. It is to be expected that a gradient of CaCl_2_ will not cause parallel substitutions in both monomers, i.e. generate asymmetry. Such may generate mixed enzymatic properties as complex as those presently found during the simultaneous reconstitution of partially demetalated TNAP with mixtures of Zn^2+^ and Ca^2+^, respectively, or Zn^2+^, Ca^2+^ and Mg^2+^. In case of active site asymmetry, cross-occupation of binding sites by the “wrong” metal may generate response profiles, hardly predicted by more straightforward approaches, based on preloaded TNAP in equilibrium conditions. Since each TNAP dimer needs to accommodate 4 metal ions in M1 and M2, before reaching symmetry, some activity measurements in intermediate stages may reflect such more complex behavior. Presently, such conditions were met during the interaction of Zn^2+^-TNAP with 10 and 20 mM CaCl_2_, modulating TNAP-activity in a time-dependent manner. Even highly pure CaCl_2_ contains metal ion contaminants, the most abundant one Sr^2+^, a potent TNAP activator [24]. Inclusion of standardized [ZnCl_2_] could overcome this limitation, allowing proper competition and displacement studies between ZnCl_2_ and high [CaCl_2_] at pH 7.4.

Medial vascular calcification is associated with chondrocyte transdifferentiation and expression of TNAP [33]. It is to be expected that also in this environment, where a gradient of calcium builds up, TNAP will be gradually substituted with Ca^2+^ at all metal ion sites. Our work also suggests that TNAP in other Ca^2+^-rich environments can act as Ca^2+^/Zn^2+^-TNAP, e.g. regulating the hydrolysis of phospholamban in the sarcoplasmic reticulum of cardiomyocytes and in skeletal muscle [34]. The injection of Zn acetate into the tail vein of mice enhanced TNAP activity in the sarcoplasmic reticulum of the cardiac sarcomere, leading to increased dephosphorylation of phospholambam. This finding supports the interpretation that also in the cardiac sarcomere TNAP activity is tempered by high prevailing Ca^2+^ levels [34]. The strong correlation between the loss of TNAP activity and the accumulation of calcium during MV-mediated mineralization, observed by Genge et al. [26] can also be explained by our present findings. In these studies TNAP activity present at the site of the MV-dependent mineralization process was found to be profoundly reduced by the mineralization process, a finding that can be explained by [CaCl_2_]-dependent conversion of Ca^2+^/Zn^2+^-TNAP into Ca^2+^/Ca^2+^-TNAP.

In conclusion, our work has identified that ionized calcium supports TNAP activity in Ca^2+^-rich milieus, until very high concentrations of Ca^2+^ occupy M1 and M2 leading to greatly reduced enzymatic activity.

## Supporting Information

S1 Fig Short-term reconstitution of Zn^2+^-TNAP activity at pH 7.4. a. Progressive Zn^2+^-TNAP formation, measured from the increase of A405 nm *vs*. time during pNPP hydrolysis, after incubation of AbM2-bound TNAP with 1 mM EDTA (2 h) followed by addition of the indicated [Zn^2+^] (2 h), dissolved in Chelex-treated TBS, followed by addition of Chelex-treated pNPP (10 mM) at pH 9.8; b. Slope (first derivative) to the line for 40 μM ZnCl_2_ in Fig. 1A; c. Dose-response of Zn^2+^-TNAP formation, from plots of initial AP activity (ΔA405 nm/20 min) *vs*. the indicated [Zn^2+^]; the red line represents the corresponding AP activity for native non-EDTA treated AbM2-boundTNAP. Results are representative of 3 independent experiments.
(TIF)

S2 Fig Activation and inhibition of EDTA-treatedTNAP by CaCl_2_, at pH 9.8. a. Progressive activation and inhibition of AbM2-bound EDTA-treated TNAP in the absence (left panel) and presence (right panel) of 2 μM ZnCl_2_, measured from its activity at A405 nm *vs*. time in Chelex-treated pNPP (10 mM) at pH 9.8; Note the curvi-linearity at low [CaCl_2_] and linearity at 5 and 10 mM CaCl_2_ respectively (left panel); the maximal activity (“max”) represents activity of fully metalated TNAP in pNPP, pH 9.8, measured in the presence of 20 μM ZnCl_2_ and 1 mM MgCl_2_; b. Dose-response of TNAP activation and inhibition, from plots of AP activity (ΔmA405nm/min calculated between 60–90 min) *vs*. the indicated [CaCl_2_] (left panel) or [MgCl_2_] (right panel); ●: absence of ZnCl_2_; ■: 2 μM ZnCl_2_ mixed with CaCl_2_; μ: 2 μM ZnCl_2_ mixed with MgCl_2_. *Insert*: one-site binding model fit for the ascending limb, without added Zn^2+^. Results are representative of 3 independent experiments.
(TIF)

S3 Fig Production and stability of TNAP M4 mutants. a. Western blots of TNAP, PLAP and their mutants, after purification from COS-1 cellular medium, via AbM2 detection; b. Heat inactivation curves of PLAP and the indicated mutants, plotted as residual activity after 10 min incubation at the indicated temperature; c. Heat inactivation curves of TNAP and the indicated mutants, plotted as residual activity after incubation for the indicated time interval at 56°C in TBS. Results are representative of 3 independent experiments.
(TIF)

S4 Fig Inhibition kinetics of TNAP by MgCl_2_ and CaCl_2_. **a**. Dose-response of Zn^2+^-TNAP inhibition by high [MgCl_2_] (0–20 mM) at pH 7.4; AP activity was measured as mean mA405nm/min in steady-state (between 60–90 min) (left panel); tracings of TNAP activity vs. time, in the presence of the indicated medium to high [MgCl_2_] at pH 7.4, illustrating a slight deflection in hydrolysis rate after 60 min (solid line *vs*. dotted line) (right panel); b. Similar tracings, in the presence of the indicated medium to high [CaCl_2_], illustrating clear deflection in hydrolysis rate after 50 min (left panel); kinetics of TNAP inactivation by high [CaCl_2_], at pH 9.8, reaching steady-state after 10 min (right panel). Activities were measured in Chelex-treated pNPP (1 mM); results represent mean ± SD for 3 identical experiments.
(TIF)

S5 Fig Impact of pNPP concentration on affinity assessment at pH 7.4. Dose-response of generated AP activity (mean mA405nm/min) in steady-state (between 60–90 min) for increasing [MgCl_2_] (a) and [CaCl_2_] (b) at identical AbM2-bound [Zn^2+^-TNAP]; activities were measured in Chelex-treated pNPP (1 mM or 10 mM as indicated) at pH 7.4. Results represent mean ± SD for 3 identical experiments.
(TIF)

S1 File Data Supplement. Sequence of the primers used for site-directed mutagenesis and presentation of the results and discussion of the data reported in the 5 supplemental figures.
(DOCX)
