# Supplementary figures and images for: Functional Significance of Calcium Binding to Tissue-Nonspecific Alkaline Phosphatase
Source: PLoS One. 2015 Mar 16;10(3):e0119874. doi: 10.1371/journal.pone.0119874 (PMC4361680; doi:10.1371/journal.pone.0119874)

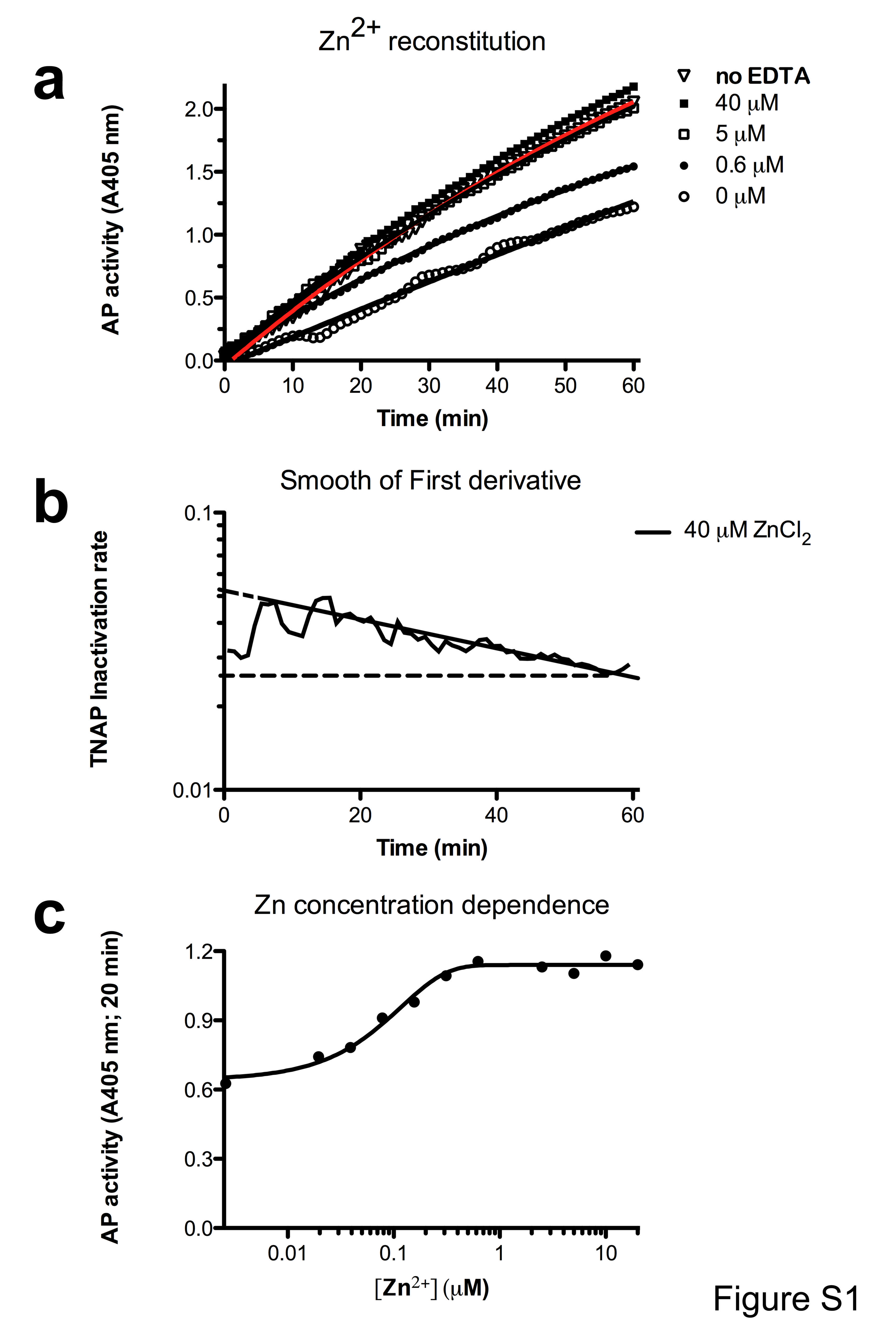

Supplement: S1 Fig — a. Progressive Zn2+-TNAP formation, measured from the increase of A405 nm vs. time during pNPP hydrolysis, after incubation of AbM2-bound TNAP with 1 mM EDTA (2 h) followed by addition of the indicated [Zn2+] (2 h), dissolved in Chelex-treated TBS, followed by addition of Chelex-treated pNPP (10 mM) at pH 9.8; b. Slope (first derivative) to the line for 40 μM ZnCl2 in Fig. 1A; c. Dose-response of Zn2+-TNAP formation, from plots of initial AP activity (ΔA405 nm/20 min) vs. the indicated [Zn2+]; the red line represents the corresponding AP activity for native non-EDTA treated AbM2-boundTNAP. Results are representative of 3 independent experiments. (TIF) [file pone.0119874.s001.tif]

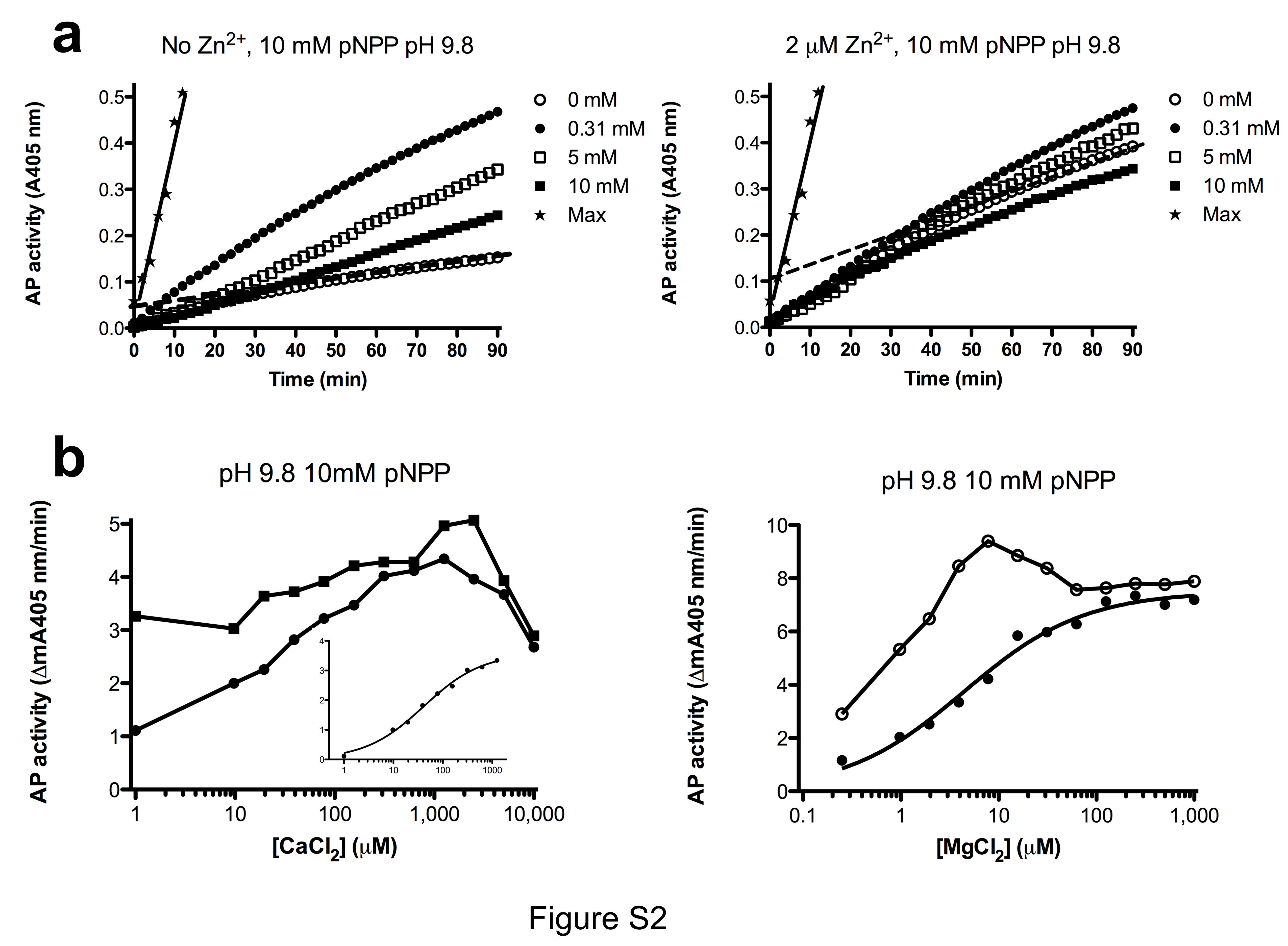

Supplement: S2 Fig — a. Progressive activation and inhibition of AbM2-bound EDTA-treated TNAP in the absence (left panel) and presence (right panel) of 2 μM ZnCl2, measured from its activity at A405 nm vs. time in Chelex-treated pNPP (10 mM) at pH 9.8; Note the curvi-linearity at low [CaCl2] and linearity at 5 and 10 mM CaCl2 respectively (left panel); the maximal activity (“max”) represents activity of fully metalated TNAP in pNPP, pH 9.8, measured in the presence of 20 μM ZnCl2 and 1 mM MgCl2; b. Dose-response of TNAP activation and inhibition, from plots of AP activity (ΔmA405nm/min calculated between 60–90 min) vs. the indicated [CaCl2] (left panel) or [MgCl2] (right panel); ●: absence of ZnCl2; ■: 2 μM ZnCl2 mixed with CaCl2; μ: 2 μM ZnCl2 mixed with MgCl2. Insert: one-site binding model fit for the ascending limb, without added Zn2+. Results are representative of 3 independent experiments. (TIF) [file pone.0119874.s002.tif]

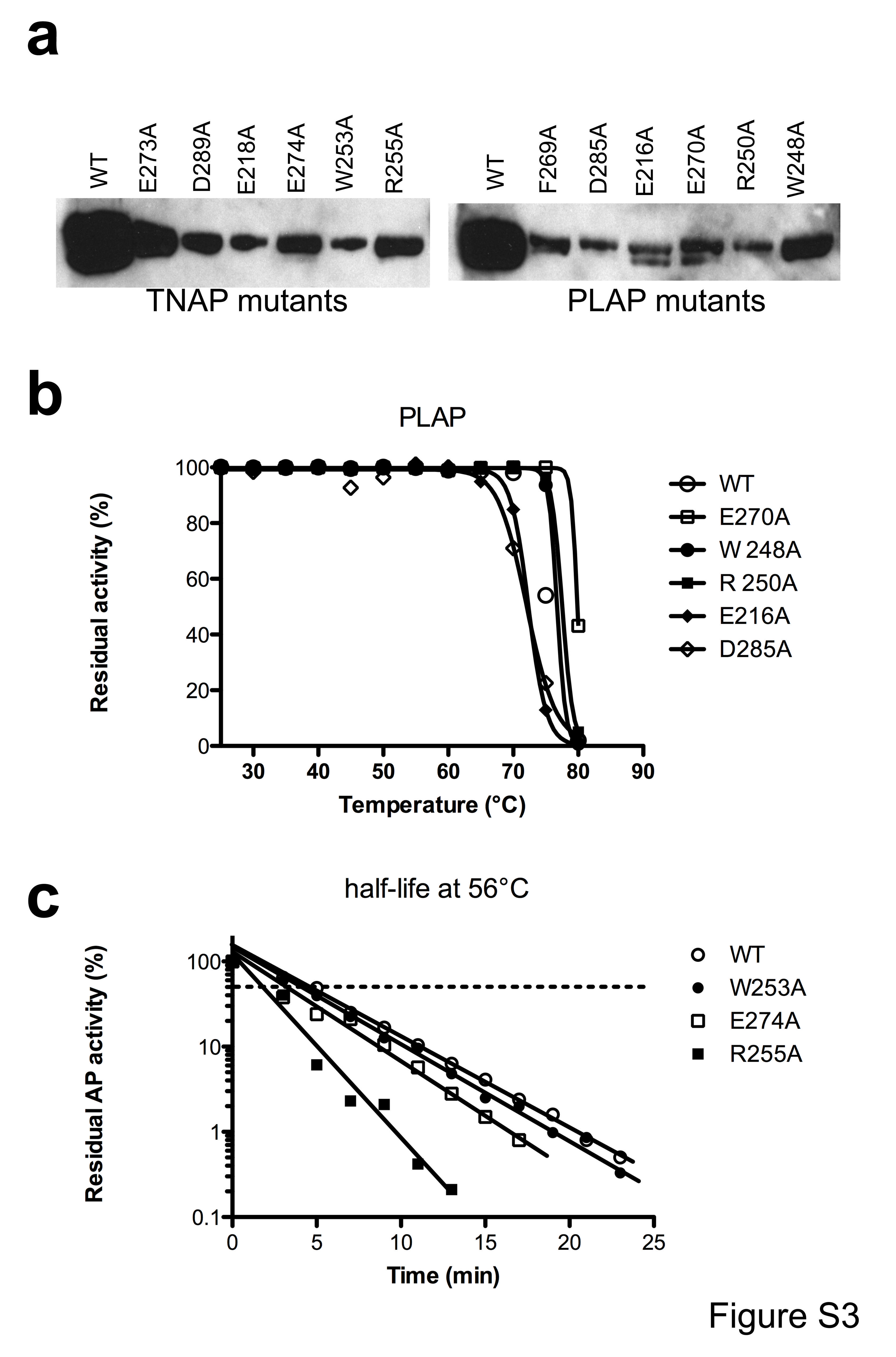

Supplement: S3 Fig — a. Western blots of TNAP, PLAP and their mutants, after purification from COS-1 cellular medium, via AbM2 detection; b. Heat inactivation curves of PLAP and the indicated mutants, plotted as residual activity after 10 min incubation at the indicated temperature; c. Heat inactivation curves of TNAP and the indicated mutants, plotted as residual activity after incubation for the indicated time interval at 56°C in TBS. Results are representative of 3 independent experiments. (TIF) [file pone.0119874.s003.tif]

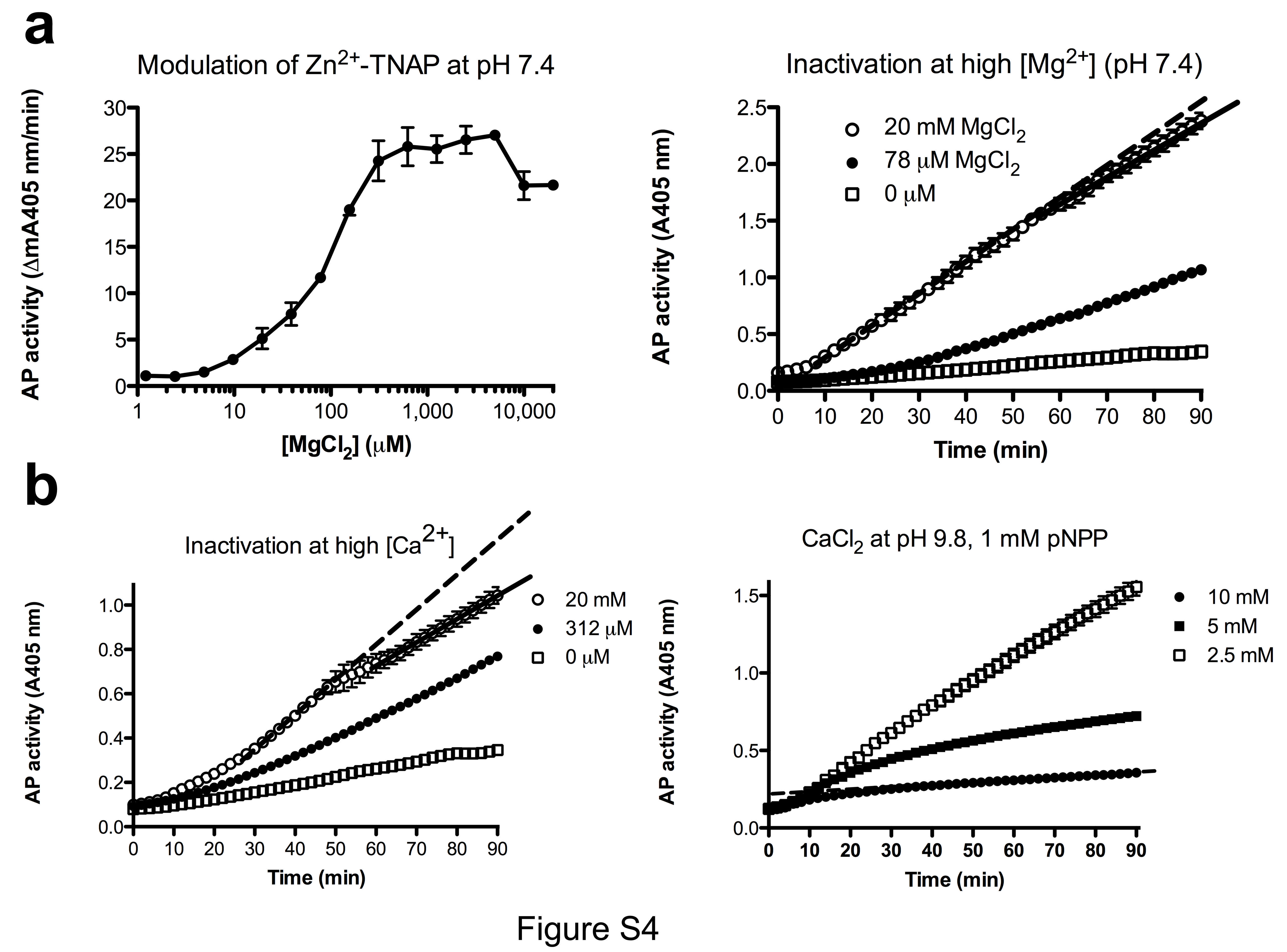

Supplement: S4 Fig — a. Dose-response of Zn2+-TNAP inhibition by high [MgCl2] (0–20 mM) at pH 7.4; AP activity was measured as mean mA405nm/min in steady-state (between 60–90 min) (left panel); tracings of TNAP activity vs. time, in the presence of the indicated medium to high [MgCl2] at pH 7.4, illustrating a slight deflection in hydrolysis rate after 60 min (solid line vs. dotted line) (right panel); b. Similar tracings, in the presence of the indicated medium to high [CaCl2], illustrating clear deflection in hydrolysis rate after 50 min (left panel); kinetics of TNAP inactivation by high [CaCl2], at pH 9.8, reaching steady-state after 10 min (right panel). Activities were measured in Chelex-treated pNPP (1 mM); results represent mean ± SD for 3 identical experiments. (TIF) [file pone.0119874.s004.tif]

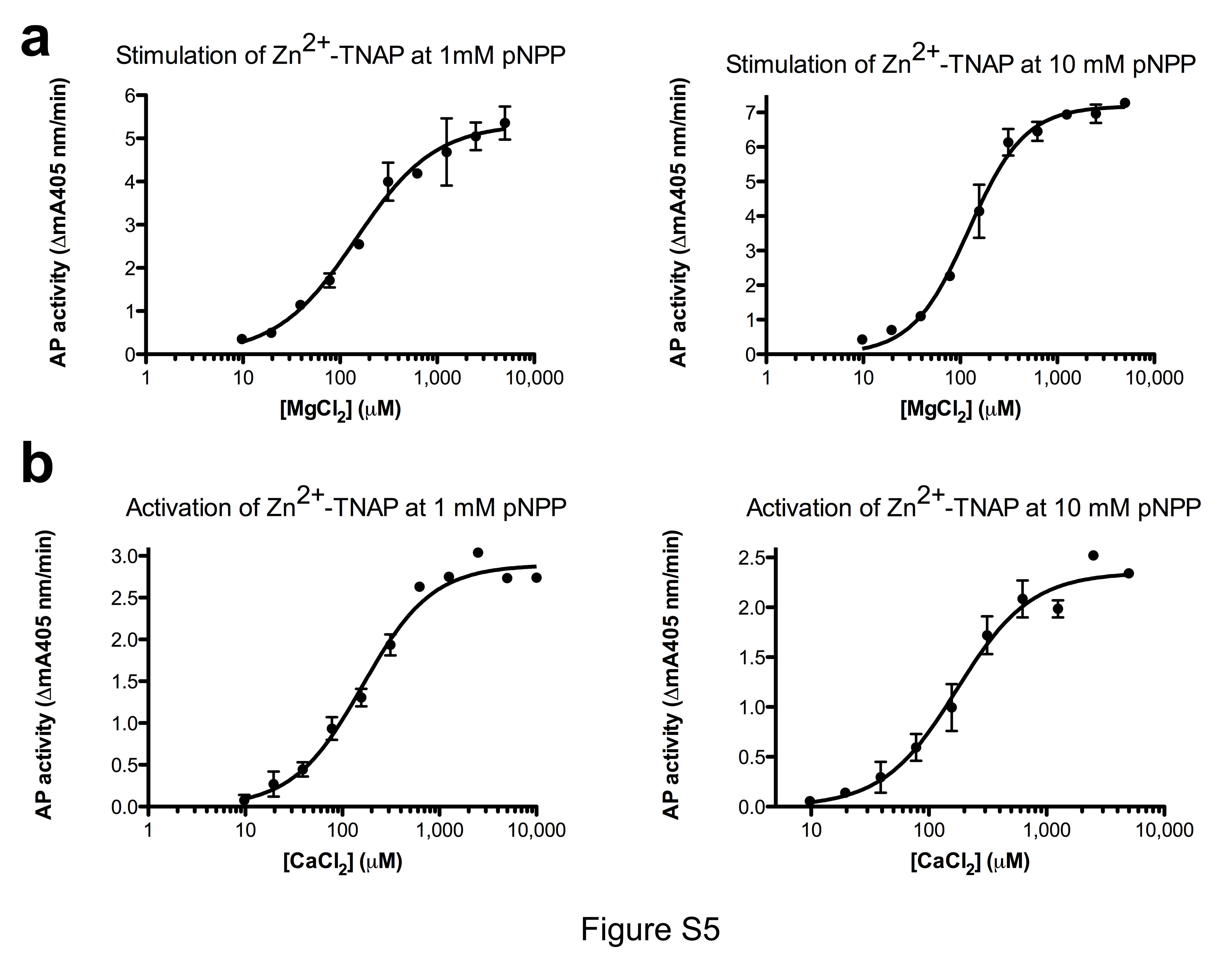

Supplement: S5 Fig — Dose-response of generated AP activity (mean mA405nm/min) in steady-state (between 60–90 min) for increasing [MgCl2] (a) and [CaCl2] (b) at identical AbM2-bound [Zn2+-TNAP]; activities were measured in Chelex-treated pNPP (1 mM or 10 mM as indicated) at pH 7.4. Results represent mean ± SD for 3 identical experiments. (TIF) [file pone.0119874.s005.tif]
