# Supplementary material for: Functional Significance of Calcium Binding to Tissue-Nonspecific Alkaline Phosphatase
Source: PLoS One. 2015 Mar 16;10(3):e0119874. doi: 10.1371/journal.pone.0119874 (PMC4361680; doi:10.1371/journal.pone.0119874)
Supplement: S1 File — Sequence of the primers used for site-directed mutagenesis and presentation of the results and discussion of the data reported in the 5 supplemental figures. (DOCX) [file pone.0119874.s006.docx]

**Data supplement**

**Materials and Methods**

**Table A. Primers used for site-directed mutagenesis in this study**

| Mutant | Primer sequence (5′­–3′) |
| --- | --- |
| **PLAP** | |
| Calcium-site Mutants | |
| E216A | AACCCCAGACCCTGCGTACCCAGATGACTAC |
|  | GTAGTCATCTGGGTACGCAGGGTCTGGGGTT |
| F269A | CCATCTCATGGGTCTCGCTGAGCCTGGAGACATG |
|  | CATGTCTCCAGGCTCAGCGAGACCCATGAGATGG |
| E270A | CTCATGGGTCTCTTTGCGCCTGGAGACATGAAATAC |
|  | GTATTTCATGTCTCCAGGCGCAAAGAGACCCATGAG |
| D285A | GACTCCACACTGGCCCCCTCCCTGATG |
|  | CATCAGGGAGGGGGCCAGTGTGGAGTC |
| Peripheral-site Mutants | |
| W248A | GTGCCCGGTATGTGGCGAACCGCACTGAGC |
|  | GCTCAGTGCGGTTCGCCACATACCGGGCAC |
| R250A | CGGTATGTGTGGAACGCCACTGAGCTCATGCAGGC |
|  | GCCTGCATGAGCTCAGTGGCGTTCCACACATACCG |
| **TNAP** | |
| Calcium-site Mutants | |
| E218A | Gaataaaactgatgtggcgtatgagagtgac |
|  | GTCACTCTCATACGCCACATCAGTTTTATTC |
| D289A | gaacaacgtgacggccccgtcactctccg |
|  | CGGAGAGTGACGGGGCCGTCACGTTGTTC |
| F273A | cctattgggtctcgccgagccgggggac |
|  | GTCCCCCGGCTCGGCGAGACCCAATAGG |
| E274A | cctattgggtctcttcgcgccgggggacatgc |
|  | GCATGTCCCCCGGCGCGAAGAGACCCAATAGG |
| Peripheral-site Mutants | |
| W253A | CCCACTTCATCGCGAACCGCACGGAACTCC |
|  | GGAGTTCCGTGCGGTTCGCGATGAAGTGGG |
| R255A | CTTCATCTGGAACGCCACGGAACTCCTGACC |
|  | GGTCAGGAGTTCCGTGGCGTTCCAGATGAAG |

Underlined nucleotides indicate mutated sites

**Results and discussion**

**Role of Zn^2+^ in TNAP activity**

M3 saturation with Mg^2+^ leads to potent allosteric TNAP activation [1]. To study the role of Ca^2+^ in M1 to M4, we developed two reproducible approaches to achieve reversible demetalation via EDTA-treatment and remetalation with Zn^2+^, Mg^2+^, Ca^2+^ or their combinations. To this end, FLAG-tagged TNAP (mutants) was adhered to microtiter plates coated with AbM2, which procedure enabled the use of soluble EDTA to complex and remove dissociated metal ions from AbM2-bound TNAP (mutant). AP apoenzymes are easily irreversibly inactivated [2] and metal ion removal with chelators favors irreversible denaturation. Our preliminary measurements showed that Zn^2+^ and Mg^2+^ indeed acted as structural determinants controlling human TNAP stability. Therefore, in most applications AbM2-immobilized TNAP was demetalated for a maximum of 2h, in the presence of 1 mM EDTA to preclude dissociated Zn^2+^ and Mg^2+^ from rebinding, after which activity was measured directly with pNPP supplemented with various metal ion concentrations, or indirectly after a 2 h metal ion loading step. In these conditions, demetalation is not complete, causing some residual baseline activity, shown in different figures. In a second approach, AbM2-bound TNAP was incubated overnight at room temperature with 250 μM EDTA, in the presence of 0.1% hSA, which procedure allowed preparation of AbM2-bound holo-TNAP, be it at the cost of some 15-20% irreversible protein denaturation (see below).

Thus, Fig. S1a in SI file shows how treatment with 1 mM EDTA for 2h did not fully eradicate TNAP activity, measured *vs.* time with chelex-treated 10 mM pNPP in 1 M DEA buffer, pH 9.8, in the absence of ZnCl_2_ (0 μM Zn^2+^). However, pre-loading with ZnCl_2_ up to 40 μM (2 h) dose-dependently raised TNAP activity to a level measured in the non-EDTA treated control, in the absence of added ZnCl_2_ or MgCl_2_ (Fig. S1a in SI File, red line). Hence, full reconstitution of TNAP occurred between 5-40 μM Zn^2+^. However, a progressive decline of TNAP activity, starting after 10 min of incubation at pH 9.8 was noted over time in all cases, also in the presence of Zn^2+^ (see also Fig. S2 in SI file). This activity drop corresponded to a monophasic disappearance curve (calculated as the slope of the tracings in Fig. S1a in SI file *vs.* time), with a half-life of around 1 h (Fig. S1b in SI file). This drop illustrates that, in the absence of M3 occupation, Zn^2+^-TNAP is not very stable at pH 9.8 at room temperature and that the loss of activity does not result from dissociation of bound Zn^2+^, but from the lack of allosteric enzyme stabilization (see Figs. 1 and 2). A tentative dose-response curve, calculated from the uncorrected A405 nm measurements over the first 20 min, yielded a dose-response curve, with plateau starting at 0.5 μM ZnCl_2_ and a K_d_ = 0.12 ± 0.2 μM for Zn^2+^ loading at physiological pH. We concluded that [ZnCl_2_] = 2 μM sufficed to just saturate M1 and M2 in TNAP at pH 9.8 and that [ZnCl_2_] = 20 μM amply sufficed to preload TNAP at physiological pH.

**Ca^2+^ binding to EDTA-treated TNAP at pH 9.8**

The curvilinear tracing of the baseline activity in Fig. S2a in SI fileconfirmed that at pH 9.8, TNAP is unstable without stabilizing metal ions in the pNPP substrate, yet reaches a near-steady-state between 60-90 min. TNAP baseline activity is measurable in this reference interval (0.77 mA405nm/min) after EDTA pre-treatment (Fig. S2a in SI file). In comparison to the maximal TNAP activity for non-EDTA treated Mg^2+^/Zn^2+^-TNAP (measured with 1 mM MgCl_2_ in pNPP substrate, yielding constant ΔA405nm/time = 42 mA405nm/min), CaCl_2_ progressively activated TNAP to a maximum of 4 mA405nm/min (60-90 min interval) at [CaCl_2_] = 1.25 mM. At higher [CaCl_2_], TNAP was dose-dependently inhibited, reaching a minimum of 2.8 mA405nm/min at 10 mM CaCl_2_. Or, at 0-1.25 mM, CaCl_2_ caused mild TNAP activation, without increasing the stability of TNAP at pH 9.8 to a maximal activity equal to 9.5% of that of Mg^2+^/Zn^2+^-TNAP (Fig. S2a in SI File). At higher concentrations, CaCl_2_ reduced activity but increased the stability of the resulting Ca^2+^/Ca^2+^-TNAP (constant ΔA405nm/time), resulting in an enzyme with an activity of 6.7% of that of Mg^2+^/Zn^2+^-TNAP ( Fig. S2a in SI file). Corresponding plots of activities *vs.* [CaCl_2_], derived for the reference interval (60-90 min) showed a curve with an ascending limb (Fig. S2b in SI file, left panel), described by a calculated K_d_ = 45 ± 7.7 μM (see fit for this rise in Fig. S2b in SI file, insert), a value 4-5 fold lower than that derived from Fig. 2b (two-tailed p<0.0001).

In parallel, we incubated increasing [MgCl_2_] with EDTA pre-treated TNAP. These incubations resulted in mild and progressive TNAP activation (increasing slope over time, not shown), as expected in the presence of allosteric enzyme stabilization and activation (see Fig. 1). Corresponding plots of activities *vs.* [MgCl_2_] (reference interval 60-90 min) resulted in a saturation curve (Fig. S2b in SI file, right panel) with a calculated K_d_ = 4.4 ± 0.7 μM, identical to that derived from Fig. 2b and compatible with Mg^2+^ saturation of M3 in EDTA-treated TNAP, containing some residual Zn^2+^, bound at M1 and M2. The additional inclusion of 2 μM Zn^2+^ in the substrate influenced this picture in three ways: 1. The baseline activity rose 3-fold, consistent with formation of additional Zn^2+^-TNAP; 2. More potent activation of the additional Zn^2+^-TNAP occurred at low [MgCl_2_] (0-10 μM); 3. Higher [MgCl_2_] (10-100 μM) competed with soluble Zn^2+^ for binding to M1 and M2, reducing activity to that in the absence of added ZnCl_2_. These findings illustrated how soluble Mg^2+^ and Zn^2+^ compete for binding to M1 and M2, at considerably lower [Mg^2+^] than required for the displacement of M1- and M2-bound Zn^2+^ by [Mg^2+^] > 1 mM (Fig. 3b, left panel).

Addition of 2 μM Zn^2+^ to each [CaCl_2_] in the substrate showed a mixed profile, in which the 3-fold increase in TNAP activity by 2 μM Zn^2+^ (no CaCl_2_) gradually faded with increasing [CaCl_2_] to 1 mM. This profile can be understood from the lower affinity of Ca^2+^ for M3 (Figs. 2 and 3) and by its competition with soluble Zn^2+^ for binding to M1 and M2, in analogy with the profile for Mg^2+^ (Fig. S2b in SI file, right panel). Hence, the Zn^2+^-mediated stimulation to a maximum (60-90 min) of 3.2 mA405nm/min (a 3-fold increase from baseline), only rises further to 4.9 mA405nm/min, at [CaCl_2_] = 1.25 mM. High CaCl_2_ concentrations were inhibitory and reduced TNAP activity below that achieved for 2 μM Zn^2+^ alone, to 2.6 mA405nm/min at 10 mM (60-90 min), i.e. to a value comparable to that for 10 mM CaCl_2_, in the absence of ZnCl_2_ (Fig. S2b in SI file). In addition, the more complex kinetic activation profiles in Fig. S2a in SI file, right panel suggest longer incubation times are required to reach steady-state. These findings illustrated that Ca^2+^ and Zn^2+^ can both bind to M1, M2 and M3, although Ca^2+^ does so with a lower affinity than Mg^2+^. Binding of Ca^2+^ to M3 did not stabilize TNAP, but at high concentrations, where also M1 and M2 were saturated, allosteric stabilization was observed. However, the bi- to triphasic dose-response curves for Ca^2+^ and Mg^2+^ at pH 9.8 confirmed that Ca^2+^ and Mg^2+^ not only compete with Zn^2+^ for binding to M1 and M2, but at high concentrations (>1 mM) also displace Zn^2+^ from M1 and M2, generating an enzyme (Ca^2+^/Ca^2+^-TNAP) with low activity (6.7% of activity for Mg^2+^/Zn^2+^-TNAP).

**Ca^2+^ binding to EDTA-treated TNAP at pH 7.4**

In contrast to the potent TNAP activation by very high [CaCl_2_] and [MgCl_2_] at pH 9.8, also at pH 7.4, 10 and 20 mM CaCl_2_ (Fig. 7b) and MgCl_2_ (Fig. S4a in SI File) were weakly disruptive for the Zn^2+^-TNAP activity. Therefore, similarly to Figs. S1 and S2 in SI File, TNAP activity was analyzed kinetically, in the presence of 20 mM CaCl_2_ or MgCl_2_. Fig. S4a in SI File, right panel shows a minor deflection from linearity in the presence of 20 mM MgCl_2_, starting after about 1 h, but Fig. S4b in SI File, left panel shows how 20 mM CaCl_2_ triggers a time-controlled effect: up to about 30 min, the activity of Zn^2+^-TNAP is progressively accelerated, as a result of Ca^2+^ binding to M3. It then reaches a steady-state for about 20 min, after which a transition occurs to a lower activity state, over an interval of 10-15 min, remaining stable over the remainder of the observation (constant slope of A405nm *vs*. time). The rate of this slope equaled 10.5 mA405nm/min, compared to 16 mA405nm/min for the preceding ascending limb, i.e. a drop of about 34% in a new steady-state. This figure illustrates the slow kinetics of Ca^2+^ binding to M1 and M2 and it effect on TNAP activity, in comparison to the potent and fast inactivation by 10 mM CaCl_2_ at pH 9.8 (Fig. S4b in SI File, right panel).

**Role of pNPP concentration at pH 7.4**

Consistent with a preponderance of HPO4-- ions at pH 7.4, determination of the Kd for binding of Mg2+ or Ca2+ to TNAP is hardly dependent on [pNPP]. Fig. S5 in SI Fileshows similar plateaus at 1 and 10 mM pNPP, a 2.5-fold difference being maintained for saturation by Ca2+ and Mg2+ respectively. In this comparison, saturation for Mg2+ is described by Kd = 146 ± 26 μM at 1 mM pNPP and Kd = 122 ± 7 μM at 10 mM pNPP. Likewise, saturation for Ca2+ is described by Kd = 161 ± 15 μM at 1 mM pNPP and Kd = 173 ± 25 μM at 10 mM pNPP. These measurements confirm that the affinity of Mg2+ for TNAP is much lower at pH 7.4 than at pH 9.8, but that the affinities describing the binding of Ca2+ are not so much different between both pHs. At physiological pH, Ca2+ has a relative advantage over Mg2+ in allosterically activating TNAP, notwithstanding that TNAP has a lower catalytic efficiency at pH 7.4 than at pH 9.8 [3].

# References

1. Cathala G, Brunel C. Bovine kidney alkaline phosphatase. Catalytic properties, subunit interactions in the catalytic process, and mechanism of Mg2+ stimulation. J Biol Chem. 1975; 250: 6046-6053.
2. Sorimachi K. Activation of alkaline phosphatase with Mg2+ and Zn2+ in rat hepatoma cells. Accumulation of apoenzyme. J Biol Chem. 1987; 262: 1535-1541.
3. Hoylaerts MF, Ding L, Narisawa S, Van Kerckhoven S, Millán JL. Mammalian alkaline phosphatase catalysis requires active site structure stabilization via the N-terminal amino acid microenvironment. Biochemistry. 2006; 45: 9756-9766.
